# The Ascomycete *Verticillium longisporum* Is a Hybrid and a Plant Pathogen with an Expanded Host Range

**DOI:** 10.1371/journal.pone.0018260

**Published:** 2011-03-24

**Authors:** Patrik Inderbitzin, R. Michael Davis, Richard M. Bostock, Krishna V. Subbarao

**Affiliations:** Department of Plant Pathology, University of California Davis, Davis, California, United States of America; Duke University Medical Center, United States of America

## Abstract

Hybridization plays a central role in plant evolution, but its overall importance in fungi is unknown. New plant pathogens are thought to arise by hybridization between formerly separated fungal species. Evolution of hybrid plant pathogens from non-pathogenic ancestors in the fungal-like protist *Phytophthora* has been demonstrated, but in fungi, the most important group of plant pathogens, there are few well-characterized examples of hybrids. We focused our attention on the hybrid and plant pathogen *Verticillium longisporum*, the causal agent of the Verticillium wilt disease in crucifer crops. In order to address questions related to the evolutionary origin of *V. longisporum*, we used phylogenetic analyses of seven nuclear loci and a dataset of 203 isolates of *V. longisporum*, *V. dahliae* and related species. We confirmed that *V. longisporum* was diploid, and originated three different times, involving four different lineages and three different parental species. All hybrids shared a common parent, species A1, that hybridized respectively with species D1, *V. dahliae* lineage D2 and *V. dahliae* lineage D3, to give rise to three different lineages of *V. longisporum*. Species A1 and species D1 constituted as yet unknown taxa. *Verticillium longisporum* likely originated recently, as each *V. longisporum* lineage was genetically homogenous, and comprised species A1 alleles that were identical across lineages.

## Introduction

Hybridization plays a central role in plant evolution. More than 10% of currently existing species might be hybrids [1], which includes many of our most important crops such as wheat, oat, cotton, coffee and canola [2]. Hybrids often exhibit new or extreme phenotypes with respect to their parents, and hybrid vigor or heterosis in cultivated crops is widely exploited.

Whereas hybridization in plants has been well studied, relatively little is known about hybrids in fungi. Historically, there have been few reports of fungal hybrids inferred from credible morphological and genetic evidence [3], [4], [5], [6]. The advent of molecular phylogenetics has greatly improved our ability to detect hybrids [7], and has resulted in increased numbers of documented examples [8], [9], [10], [11], [12], [13], [14], [15], [16], [17], [18], [19].

The consequences of hybridization at the molecular level in fungi are unknown, but in plants they include both genomic and epigenetic changes resulting in the alteration of gene expression and the evolution of new phenotypes [1], [2], [20], [21]. In natural fungal hybrids, both morphological and life style changes have been observed. Hybrids may display intermediate parental morphology [3], [4], [5], [9] and fermentation properties [10], a switch from pathogen to mutualist [22], an increase in virulence [3], a decrease in fitness [13] and a new host range with respect to their parents [3]. Some of these changes are also observed in artificial hybrids [16], [23].

Most known cases of fungal hybrids involve plant pathogens. It has been suggested that new plant pathogens with increased virulence and extended host range could arise through hybridization between formerly geographically separated species, brought into contact through human activities such as global trade [24], [25].

In fungi, there are only a limited number of examples of natural hybrid pathogens with increased virulence and extended host range. These include *Melampsora* x *columbiana* G. Newc., a hybrid between *M. medusae* and *M. occidentalis* that became the dominant rust on hybrid poplar *Populus trichocarpa* x *P. deltoides* in the Pacific Northwest in 1998 [3], as well as *V. longisporum*, a pathogen of crucifers. Molecular evidence suggests that *V. longisporum* is an allopolyploid, possibly between *V. dahliae* and a species related to *V. albo-atrum* [26]. *Verticillium longisporum* is the cause of Verticillium wilt of oilseed rape, a major disease in Europe [27] that is unknown in North America [28]. Even though *V. dahliae*, a relative of *V. longisporum*, is at times isolated from oilseed rape, only *V. longisporum* is capable of causing disease [29]. Both *V. longisporum* and *V. dahliae* survive as microsclerotia in the soil, which facilitates pathogen dispersal through water, farm equipment and personnel [30].

Laying the foundations for future studies on pathogenicity, virulence and host specificity in *V. longisporum*, we employed a phylogenetic approach using 203 isolates of *V. longisporum*, *V. dahliae*, *V. albo-atrum*, *V. nubilium* and *V. tricorpus*, and seven different loci, to elucidate the parental species and hybridization events involved in the evolution of *V. longisporum*.

## Results

### Allelic diversity in species of *Verticillium*

The typical ascomycete life cycle is dominated by the haploid state, but *Verticillium longisporum* is diploid, its nuclei contain approximately twice the amount of DNA as the closely related *V. dahliae* [31], [32], [33]. We found that all *V. longisporum* strains contained two alleles at each of the eight loci examined, except at the nuclear ribosomal internal transcribed spacer region (ITS) which contained a single allele, and at the *beta-tubulin* locus which was represented through an additional, paralogous copy in some isolates of *V. dahliae* and *V. longisporum*. These conclusions were derived from sequencing 225 PCR product clones from 12 different strains and all eight loci (Table S1), and PCR screening of *V. longisporum* strains using allele specific primers (Figures S1, S2, S3), followed by DNA sequencing and phylognetic analyses. All loci in *V. albo-atrum*, *V. nubilum* and *V. tricorpus* contained only a single allele, as inferred by the absence of polymorphic positions in directly sequenced PCR products.

### Mating type idiomorphs in *V. longisporum* and other species

Mating polarity in ascomycetes is determined by the mating type locus that contains alleles differing in gene content referred to as idiomorphs [34]. Idiomorphs are designated by ‘*MAT’* followed by a series of numbers that reflect idiomorph gene content. Two strains are sexually compatible if they carry either a *MAT1-1* or a *MAT1-2* idiomorph [35]. We found that *V. longisporum* strains contained two *MAT1-1* idiomorphs based on cloning and DNA sequencing (Table S1), and failed to detect *MAT1-2* using idiomorph-specific PCR screens (Figure S4). DNA sequence data obtained from *V. longisporum* strains PD342, PD348 and PD356 showed that the *V. longisporum MAT1-1* idiomorph contained *MAT1-1-1* approximately 1.5 kb upstream of *MAT1-1-2* (GenBank Accessions HQ415004 - HQ415009), as in the closely related *V. albo-atrum* (Klosterman et al., accepted for publication in *PLoS Pathogens* pending revisions).

The majority of the *V. dahliae* strains carried a *MAT1-2* idiomorph, only 11 strains were *MAT1-1* (Figure S4). DNA sequence data of the region spanning from *MAT1-1-1* to *MAT1-1-2* was obtained for the *V. dahliae MAT1-1* strains PD404, PD585 and PD617 (GenBank Accessions HQ415012, HQ415014, HQ415025), confirming the presence of *MAT1-1-1* and *MAT1-1-2* arranged as in *V. longisporum*.

*Verticillium albo-atrum* strains were found to be either *MAT1-1* or *MAT1-2*, the mating types for the remaining species could not be determined (Figure S4).

### Preliminary phylogenetic analyses

**Figure 1:**
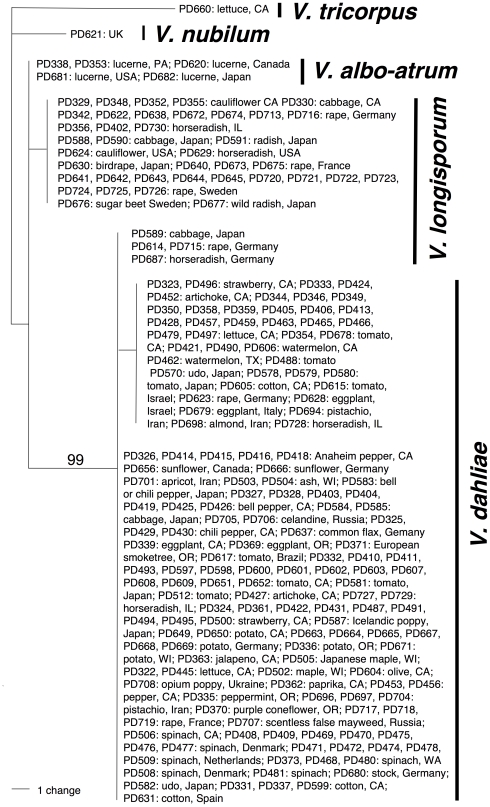
Evolutionary origins of the diploid hybrid *Verticillium longisporum* based on phylogenetic inference from ribosomal internal transcribed spacer (ITS) data comprising 203 taxa and 493 characters. Shown is the single, most parsimonious tree, 20 steps in length. Isolates are represented by a strain identifier. Hosts and geographic origins are given. Bootstrap supports above 70% are shown by the branches. Species delimitations are indicated by vertical bars on the right. The isolates fell into five different groups corresponding to the five species of *Verticillium*. The exceptions were four isolates of *V. longisporum*, *V. longisporum* strains PD589, PD614, PD687 and PD715, which grouped with *V. dahliae*.

Phylogenetic analyses based on the ITS region were used to investigate the relationships of all strains included in this study (Table S2). An alignment comprising 203 taxa and 493 characters was generated and analyzed using parsimony, resulting in one most parsimonious tree (Figure 1). The most parsimonious tree contained five different clades corresponding to the five different species included in these analyses, *Verticillium tricorpus*, *V. nubilum, V. albo-atrum*, *V. dahliae* and *V. longisporum*. The exceptions were *V. longisporum* strains PD589, PD614, PD687 and PD715, which grouped with *V. dahliae* (Figure 1). For additional details on the ITS analyses, see Table 1. The alignment was submitted to TreeBase (http://purl.org/phylo/treebase/phylows/study/TB2:S11128), the DNA sequences were deposited in GenBank. (GenBank Accessions HQ206718 - HQ206920).

**Table 1:** Statistics of the ITS, *ACT, EF, GPD, OX* and *TS* single locus datasets and the combined five-locus dataset and respective most parsimonious trees.

	Haplotypes	Characters	Variable characters	Pars info characters	MPTs: number/steps	CI/RI^b^	Clades >70% support
*ITS*	5	493	20 (4%)^a^	6 (1%)^a^	1/120	1.000/1.000	1
*ACT*	7	532	171 (32%)	53 (10%)	1/199	0.956/0.904	7
*EF*	11	600	251 (42%)	70 (12%)	6/318	0.947/0.832	9
*GPD*	9	678	131 (19%)	44 (6%)	1/165	0.909/0.741	8
*OX*	9	606	172 (28%)	64 (11%)	1/214	0.953/0.870	7
*TS*	10	591	177 (30%)	52 (9%)	35/243	0.930/0.754	8
Combined *ACT, EF, GPD, OX, TS*	21	3007	902 (30%)	283 (9%)	6/1152	0.932/0.832	14

a Percentages refer to the proportions of variable and parsimony informative characters in each dataset.

b CI: consistency index; RI: retention index.

### Phylogenetic analyses based on five protein coding genes

From each of the five clades in the ITS tree (Fig. 1), isolates were chosen according to host and geographic origin for sequencing of intron-rich portions of the five protein coding genes *actin* (*ACT*), *elongation factor 1-alpha* (*EF*), *glyceraldehyde-3-phosphate dehydrogenase* (*GPD*), *mitochondrial oxaloacetate transport protein* (*OX*) and *tryptophan synthase* (*TS*). Within *V. dahliae*, all isolates with the rare *MAT1-1* idiomorph were also selected, in *V. longisporum* all isolates grouping with *V. dahliae*, as well as all *V. longisporum* isolates with the D2 and D3 alleles which were less abundant than the D1 allele (see below for information on allelic diversity). In total, 73 taxa were chosen, which included one strain of *V. tricorpus* and *V. nubilum*, two strains of *V. albo-atrum*, 47 strains of *V. dahliae* and 22 *V. longisporum* strains. Each *V. longisporum* strain was represented by two alleles. The resulting five datasets *ACT*, *EF*, *GPD*, *OX* and *TS* were first analyzed separately using parsimony to examine congruence. Most parsimonious trees from the five analyses are shown in Figures S5, S6, S7, S8, S9. The topologies of all most parsimonious trees were identical except within the clade containing the *V. dahliae* isolates, suggesting incomplete sorting of ancestral polymorphisms or sexual recombination [36]. For more details on the single dataset analyses, see Table 1.

**Figure 2:**
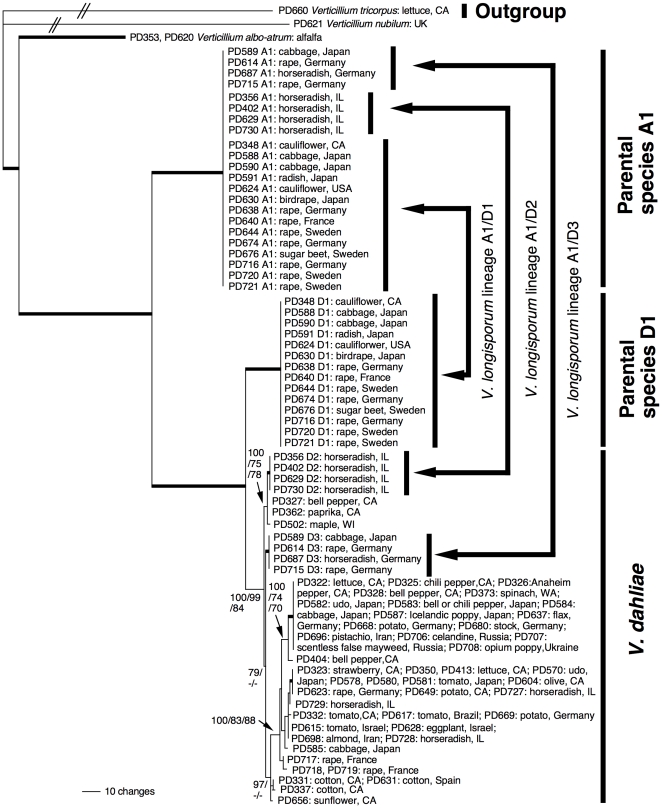
Evolutionary origins of the diploid hybrid *Verticillium longisporum*. Shown is a Bayesian consensus tree inferred from a combined *ACT*, *EF*, *GPD*, *OX*, *TS* dataset (95 taxa, 3007 characters). Isolates are represented by a strain identifier, in the case of *V. longisporum* followed by allele designations A1, D1, D2 or D3. Hosts and geographic origins are given. Species delimitations are indicated by vertical bars on the right. Values by the branches are Bayesian posterior probabilities, parsimony and likelihood bootstrap supports, in that order. Additional support values within *V. dahliae* are listed in Table S3. Branches in bold have maximal support in all analyses. The branches leading to *V. tricorpus* and *V. nubilum* are not to scale. Each *V. longisporum* isolate has two alleles that group in four different clades corresponding to three different species. One allele of each *V. longisporum* isolate groups in species A1, the second allele clusters in species D1, *V. dahliae* lineage D2 or *V. dahliae* lineage D3. *Verticillium longisporum* alleles D2 are most closely related to the free-living haploid *V. dahliae* strains PD327, PD362 and PD502. None of the other *V. longisporum* alleles group with any haploid parental isolates. Arrows on the right show the three inferred hybridization events between species A1 and species D1, *V. dahliae* lineage D2 or *V. dahliae* lineage D3, respectively, to give rise to the three lineages of *V. longisporum*, *V. longisporum* lineage A1/D1, lineage A1/D2 and lineage A1/D3. Of the three parental species, only *V. dahliae* is known.

Since the single dataset analyses did not uncover conflicts between datasets except within *V. dahliae*, the five datasets were concatenated into one alignment containing 95 taxa and 3007 characters, and analyzed using Bayesian inference, parsimony, and maximum likelihood. Bayesian inference and maximum likelihood analyses implemented a GTR+G model of DNA sequence evolution, the most appropriate model as determined by the Akaike Information Criterion in Modeltest. The Bayesian consensus tree is shown in Figure 2, rooted with *V. tricorpus* based on a study by Pantou et al. [37]. Phylogenetic analyses identified four different alleles in *V. longisporum*, alleles A1, D1, D2 and D3. As diploids, each *V. longisporum* isolate contained two alleles at each locus, allele A1 was present in all *V. longisporum* isolates, in addition to allele D1, D2 and D3, respectively. The four alleles represented four different lineages; there was no variation within lineages. Lineage A1 contained alleles A1 of all 22 *V. longisporum* isolates. Lineage D1 comprised alleles D1 of 14 *V. longisporum* isolates, lineage D2 contained alleles D2 of four *V. longisporum* isolates, and lineage D3 contained alleles D3 of the four remaining *V. longisporum* isolates. *Verticillium longisporum* lineage D2 was most closely related to the three *V. dahliae* strains PD327, PD362 and PD502, whereas *V. longisporum* lineage D3 was sister group to the remaining *V. dahliae* isolates. *Verticillium longisporum* lineages D2 and D3, as well as the *V. dahliae* isolates were sister group to *V. longisporum* lineage D1. The *V. longisporum* lineages D1, D2, D3 and the *V. dahliae* isolates were the sister group to *V. longisporum* lineage A1. The two *V. albo-atrum* isolates were monophyletic, and formed the sister group to the *V. longisporum* lineages plus the *V. dahliae* isolates. The *V. albo-atrum* and *V. dahliae* isolates, together with the *V. longisporum* lineages, were sister to *V. tricorpus* plus *V. nubilum*.

To determine which clades represented phylogenetic species, we applied the Genealogical Concordance Phylogenetic Species Recognition concept according to which well supported, terminal clades in multigene phylogenies are recognized as phylogenetic species [38]. We thus identified the following phylogenetic species: The clade with the two *V. albo-atrum* isolates, *V. longisporum* lineage A1, *V. longisporum* lineage D1 and the monophyletic group with *V. longisporum* lineages D2 and D3 as well as the *V. dahliae* isolates. The *V. longisporum* lineages D2 and D3 did not correspond to separate phylogenetic species, since they were part of a monophyletic group together with the *V. dahliae* isolates, within which there were topological conflicts between the single gene trees (Figures S5, S6, S7, S8, S9), likely indicative of sexual recombination [38]. Close affinity of *V. longisporum* lineages D2 and D3 with the *V. dahliae* isolates was supported by morphological evidence. *Verticillium dahliae* strains PD327, PD362 and PD502, the closest relatives of *V. longisporum* lineage D2, were morphologically indistinguishable from the remaining *V. dahliae* isolates. Thus, haploid isolates of the *V. longisporum* lineages D2 and D3 would be expected to belong to the morphological species *V. dahliae*. No morphological species corresponding to *V. longisporum* lineages A1 and D1 are known. Our analyses included representatives of all four haploid *Verticillium* morphological species [39], none of which grouped with *V. longisporum* lineages A1 and D1. The phylogenetic species containing the *V. albo-atrum* isolates corresponded to the morphological species *V. albo-atrum*.

The ITS tree shown in Figure 1 is not directly comparable to the Bayesian consensus tree based on five protein coding loci shown in Figure 2, since all *V. longisporum* isolates only carried a single ITS allele. *Verticillium longisporum* ITS alleles fell into two groups, correlating with the *V. longisporum* lineages D1, D2 and D3. Isolates of *Verticillium longisporum* lineages D1 and D2 had identical ITS alleles, forming a sister group to the *V. dahliae* clade in a polytomy with the *V. albo-atrum* isolates (Figure 1). ITS alleles of *V. longisporum* lineage D3 were identical to the ITS alleles of the majority of *V. dahliae* isolates.

The phylogenetic analyses using parsimony and maximum likelihood supported the results from Bayesian inference on the combined five-locus dataset. Parsimony analyses yielded six most parsimonious trees of 1152 steps each, differing from each other and the tree in Figure 2 by poorly supported branches within *V. dahliae*. See Figure 2 for parsimony branch supports, and Table 1 for additional details in the parsimony analyses. The most likely tree had a –ln likelihood score of 8851.96, and differed from the tree in Figure 2 and the most parsimonious trees by poorly supported branches within *V. dahliae* (see Figure 2 for likelihood branch supports). See Table S3 for support values within *V. dahliae* not presented in Figure 2. The five-locus alignment was submitted to TreeBase (http://purl.org/phylo/treebase/phylows/study/TB2:S11128), and the DNA sequences were deposited in GenBank (Accessions: *ACT:* HQ206921 - HQ207015; *EF*: HQ414624 - HQ414718, *GPD*: HQ414719 - HQ414813, *OX*: HQ414814 - HQ414908, *TS*: HQ414909 - HQ415003).

### Phylogenetic analyses and taxon selection for the *MAT1-1* tree

**Figure 3:**
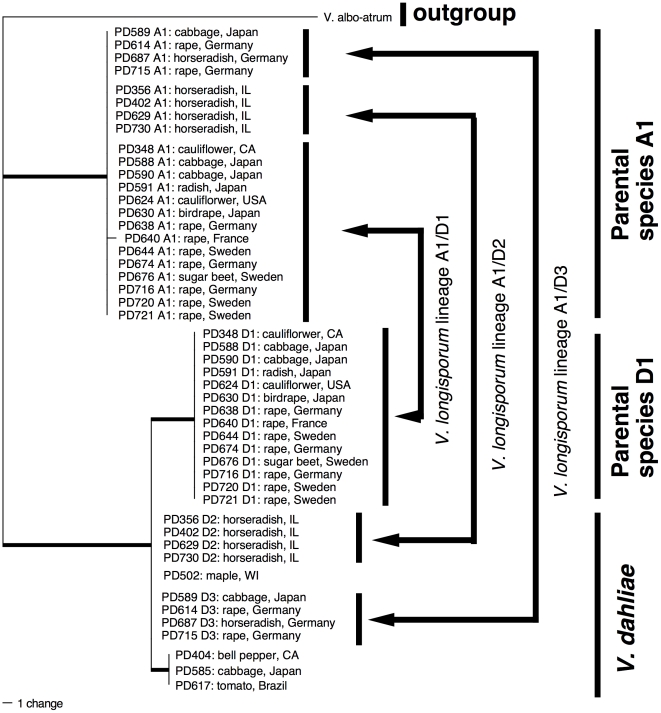
Evolutionary origins of the diploid hybrid *Verticillium longisporum* based on partial *MAT1-1* idiomorph data. Shown is a Bayesian consensus tree inferred from non-coding region between *MAT1-1-1* and *MAT1-1-2* (45 taxa, 420 characters). Taxa included were all *V. longisporum* strains used in Fig. 2, as well as a selection of *V. dahliae MAT1-1* isolates. Isolates are represented by a strain identifier, in the case of *V. longisporum* followed by allele designations A1, D1, D2 or D3. Hosts and geographic origins are given. Species delimitations are indicated by vertical bars on the right. Values by the branches are Bayesian posterior probabilities, parsimony and likelihood bootstrap supports, in that order. Branches in bold have maximal support in all analyses. Each *V. longisporum* isolate has two alleles that group in three different clades corresponding to three different species. One allele of each *V. longisporum* isolate groups in species A1, the second allele clusters in species D1 or in *V. dahliae* lineages D2/D3. Unlike in the five-locus tree in Figure 2, *V. dahliae* lineages D2 and D3 are not differentiated, otherwise results are congruent. Arrows on the right show the three inferred hybridization events between species A1 and species D1, *V. dahliae* lineage D2 or *V. dahliae* lineage D3, respectively, to give rise to the three lineages of *V. longisporum*, *V. longisporum* lineage A1/D1, lineage A1/D2 and lineage A1/D3. The *V. albo-atrum* sequence was retrieved from the BROAD website from supercontig 1_2.

Results from phylogenetic analyses based on the *MAT1-1* region (Figure 3) were congruent with results from the tree derived from five loci (Figure 2), except that *V. dahliae* lineages D2 and D3 were not resolved. All *V. longisporum* isolates in Figure 2 were included in the *MAT1-1* analyses along with the four *V. dahliae* strains PD404, PD502, PD585 and PD617, and the homologous region from the genome sequence of *V. albo-atrum* strain retrieved from the Broad Institute website. The *V. dahliae* strains were randomly chosen among the *V. dahliae* strains with *MAT1-1* with the exception of strain PD502, which was included because it was the only *V. dahliae MAT1-1* strain sharing a most recent common ancestor with a *V. longisporum* group. The *MAT* dataset consisted of 45 taxa and 420 characters from the intergenic spacer region between *MAT1-1-1* and *MAT1-1-2* (GenBank Accessions HQ415004 - HQ415049). The alignment was submitted to TreeBase (http://purl.org/phylo/treebase/phylows/study/TB2:S11128) and analyzed using Bayesian inference, parsimony, and maximum likelihood. Both the Bayesian consensus tree shown in Figure 3, the most parsimonious tree (73 steps in length) and the most likely tree (–ln likelihood  =  922.64) had identical topologies (see Figure 3 for branch support values). The K81 model of DNA sequence evolution was used for likelihood and Bayesian analyses as determined by Modeltest.

### Detection of a *beta-tubulin* paralog in *V. dahliae* and *V. longisporum*

**Figure 4:**
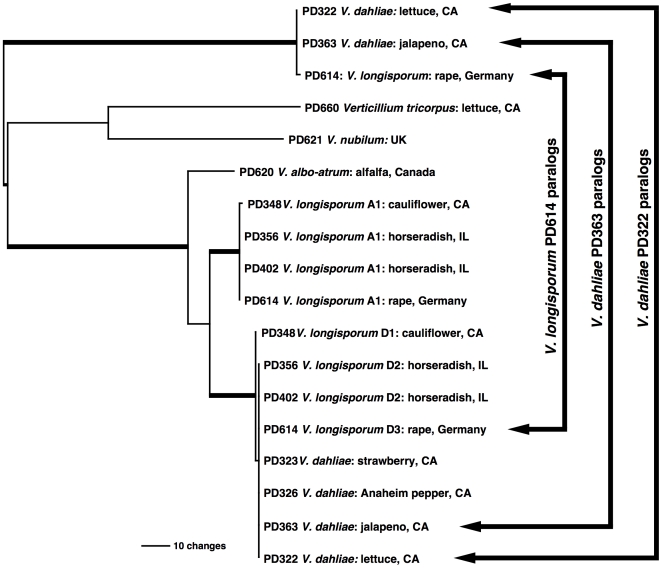
Presence of *beta-tubulin* paralogs in *V. dahliae* and *V. longisporum* isolates. Shown is a most parsimonious tree from a 18 taxa, 666 character dataset, 399 steps in length. The taxa selected represent the major clades from the five-locus tree in Figure 2. Isolates are represented by a strain identifier. Hosts and geographic origins are given. Arrows in the right mark the pairs of paralogs present in *V. dahliae* strains PD322 and PD363 as well as *V. longisporum* strain PD614. The tree suggests that the paralogs originated before speciation in *Verticillium*. No paralogs were found in any of the other taxa included in the tree, but not all taxa used in this study were investigated for the presence of *beta-tubulin* paralogs.

*Beta-tubulin* (*TUB)* has frequently been used in phylogenetic studies of fungi, including *Verticillium* [40], [41], [42], [43]. We did not use the *TUB* in this study, as initial PCR screens followed by cloning and phylogenetic analyses showed that *V. dahliae* strains PD322 and PD363, as well as *V. longisporum* strain PD614 harbored a *TUB* paralog. Phylogenetic analyses involving the *TUB* paralogs were performed on an 18 taxa, 666 character dataset using parsimony, resulting in two most parsimonious trees of 399 steps each (CI  =  0.912; RI  =  0.934) that were topologically identical on a 70% bootstrap support level. The taxa included in the *TUB* analyses represented all species and major lineages found in the phylogenetic analyses based on the combined, five-locus dataset (Figure 2). The most parsimonious *TUB* tree illustrated in Figure 4 showed that the three paralogs formed the sister group to the remaining taxa, and thus arose by gene duplication before speciation of *V. tricorpus*, *V. nubilum*, *V. albo-atrum*, *V. longisporum* and *V. dahliae*. No *TUB* paralogs were detected in any other taxa included in the tree, no other taxa included in this study were screened for *TUB* paralogs. The alignment was submitted to TreeBase (http://purl.org/phylo/treebase/phylows/study/TB2:S11128), and the DNA sequences were deposited in GenBank (Accessions JF343442 - JF343459).

## Discussion

Our results suggest that *Verticillium longisporum* is an allodiploid hybrid that originated at least three different times in independent hybridization events involving four different parental lineages representing three different species.

### *Verticillium longisporum* is diploid

Unlike the majority of ascomycetes, *Verticillium longisporum* is a stable diploid [6]. Its nuclei contain approximately twice as much DNA as the closely related *V. dahliae* [31], [32], [33], its conidia (asexual spores) are uninucleate and almost twice the size of *V. dahliae* conidia [44], [45]. Artificial diploids of *V. dahliae* have similar conidia sizes as *V. longisporum* [46], and artificial haploids of *V. longisporum* have similar conidia to wild type *V. dahliae* [6]. Auxotrophic mutants of *V. longisporum* are more challenging to obtain than in *V. dahliae* [6], [47], [48]. Our data are in agreement with *V. longisporum* being diploid. *Verticillium dahliae*, a close relative of *V. longisporum*, has six or seven chromosomes [49], [50]. We assayed six non-ribosomal loci that in *V. dahliae* are on at least five different chromosomes, and found two alleles at each locus (Figures 2 &3). Since all our strains were derived from single conidia that are uninucleate [31], [44], [45], this suggested that *V. longisporum* nuclei are diploid. All *V. longisporum* strains had just one ITS allele, consistent with the homogenizing selection pressure exerted on ribosomal gene repeats by concerted evolution. All other *Verticillium* species assayed here had just one allele at each locus, which is the norm in ascomycete fungi which are haploid.

### *Verticillium longisporum* has three different parental species

Phylogenetic analyses demonstrated that *V. longisporum* had three different parental species, species A1, species D1 and *V. dahliae*. Each species corresponded to a monophyletic group with maximal statistical support. Species A1 and species D1 did not correspond to known *Verticillium* species. We included representatives of all five *Verticillium* species [39], none of which clustered with species A1 and species D1. Blast searches at GenBank with *V. longisporum* alleles A1 and D1 did not return any 100% matches other than to *V. longisporum* sequences.

It was previously suggested that parents of *V. longisporum* included *V. dahliae*, *V. albo-atrum* and/or close relatives of the two [26], [40], [41], [45]. Our data demonstrated that the parents were *V. dahliae* and its close relative, species D1, as well as the more divergent species A1, sister taxon to species D1 plus *V. dahliae* (Figure 2). Species D1 differed from *V. dahliae* by 1.4% of all sequenced sites, and species A1 and *V. dahliae* differed by 4.4%. The phylogenetic topology together with the fixed genetic differences suggested that the three species have been distinct entities for a considerable amount of time.

Collins et al. [40] suggested that one of the *V. longisporum* parents was a diploid strain of *V. dahliae*, based on the presence of two *beta-tubulin* alleles in some strains of *V. dahliae* and *V. longisporum*. Our phylogenetic anlayses showed that *V. dahliae* and *V. longisporum* strains may contain a *beta-tubulin* paralog (Fig. 4). No other loci we examined in *V. dahliae* or in any other *Verticillium* species besides *V. longisporum* contained more than one allele. Thus, a more likely scenario for the presence of an additional *beta-tubulin* copy in some *V. dahliae* and *V. longisporum* strains is ancient duplication with subsequent differential losses during speciation in *Verticillium*.

### *Verticillium longisporum* originated at least three times

How often *V. longisporum* originated is unclear. Collins et al. [40] suggested two or possibly three different hybridization events. Using some of the same strains as Collins et al. [40], we demonstrated that *V. longisporum* originated at least three different times given our taxon sampling (Figure 2). Species A1 was involved in each hybridization event, hybridizing with species D1 resulting in *V. longisporum* lineage A1/D1, with *V. dahliae* lineage D2 resulting in *V. longisporum* lineage A1/D2, and with *V. dahliae* lineage D3 resulting in *V. longisporum* lineage A1/D3. This conclusion is derived from the observation that each *V. longisporum* strain contained a species A allele, as well as a species D1, *V. dahliae* lineage D2 or *V. dahliae* lineage D3 allele, at each non-ribosomal locus examined (Figure 2). The ITS locus was monomorphic in all *V. longisporum* strains, likely due to concerted evolution. The *V. longisporum* lineages A1/D1 and A1/D2 contained the ITS region of species A1, and all isolates of *V. longisporum* lineage A1/D3 contained *V. dahliae* ITS regions (Figure 1).

It was previously known that *V. longisporum* was genetically diverse. We obtained some groupings similar to those reported earlier, the *V. longisporum* lineage A1/D1 corresponded to Zeise and von Tiedemann's [51] group lsp and Clewes et al. 's [26] group alpha. The *V. longisporum* lineage A1/D3 corresponded to group lsp* and group beta of Zeise and von Tiedemann [51], and Clewes et al. [26], respectively. The group beta-gamma of Clewes et al. [26] corresponded to *V. longisporum* lineage A1/D2. Karapapa et al. [45] found another group, called recombinants which we could not confirm. *Verticillium longisporum* strain PD674, one of the recombinants, was a member of *V. longisporum* lineage A1/D1 (Figure 2).

Multiple evolutionary origins of hybrids in plants are well documented [52]. A fungal example includes the poplar rust *Melampsora* x *columbiana* that has evolved multiple times in different geographic areas [3].

### *Verticillium longisporum* likely did not originate through parasexual processes

It has been suggested that *V. longisporum* originated by parasexual recombination [45], which involves hyphal fusion, karyogamy, mitotic recombination and chromosome loss to restore the haploid state [53]. A parasexual origin is supported by the observation that nuclei of *V. longisporum* contain approximately double the DNA of *V. dahliae* [31], [44]. But there is considerable variation in DNA content between nuclei of different isolates, suggesting differences in chromosome content between isolates. This is exemplified by two isolates included in our study, *V. longisporum* strains PD721 and PD644, whose respective nuclear DNA contents were 0.05 and 0.075 pg DNA/nucleus [33]. Also, parasexual recombination has been observed in the laboratory within and between species of *Verticillium* [46], [54], making it an attractive hypothesis to account for the evolution of *V. longisporum*. However, our data do not support parasexual recombination. All *V. longisporum* strains we examined had two alleles at all six non-ribosomal loci (Figures 2 & 3), which suggested they are stable and have not reverted to a haploid state. This was despite the fact that 18 of our strains were isolated more than 13 years ago, the oldest 51 years ago (Table S2). Genetically stable hybrids in *Verticillium* have also been found in some laboratory experiments [55], but stability varies between parental strains even within a single species [54]. The variability of nuclear DNA content in *V. longisporum* isolates is similar to what is known from other fungi [56], including *V. dahliae* [49], [50]. Thus, the evidence at hand does not suggest that *V. longisporum* originated through parasexual recombination. Hyphal fusion between strains of different species, followed by nuclear fusion leading to the creation of a stable, diploid nucleus, seems more plausible.

An alternative explanation for the origin of *V. longisporum* is sexual recombination without separation of homologous chromosomes. However, this seems less likely. In ascomycetes, sexual recombination is initiated when two individuals of opposite mating types fuse. We showed that all *V. longisporum* isolates lacked the mating type *MAT1-2* (Figure S4), but carried two idiomorphs (alleles) of the other mating type, *MAT1-1*, one from each parent (Figure 3). In addition, no sexual state has ever been detected in any species of *Verticillium*, including *V. dahliae* [57].

### *Verticillium longisporum* originated on unusual host for both parents

Compared with animals and plants, not much is known about the evolutionary origins of fungal hybrids. One of the best-studied groups of fungal hybrids includes *Neotyphodium*, a genus of grass endophytes. *Neotyphodium* hybrids often have different hosts than their parents [22], [58]. This suggested that hybridizations occurred on unusual hosts for both parents, increasing the chances for the hybrid to outcompete its parents [58]. A similar scenario might be playing out in *Verticillium* [59]. Nothing is known about the life styles of two of the *V. longisporum* parents, species A1 and species D1, none of which has ever been found. Species A1 and species D1 might be associated with non-agricultural hosts or live as saprobes which would explain why they have been overlooked so far, or they might be extinct. The life style of species A1 and species D1 might be similar to *V. nubilum*, another potentially saprobic species in *Verticillium*. *Verticillium nubilum* was originally isolated from potato where it failed to induce disease symptoms [60]. *Verticillium dahliae*, the third parent, has a wide host range and infects many different crops [30], which in general excludes crucifers with some exceptions [61], [62]. The host range of *V. longisporum* is centered on crucifers [61], [63], but infection of a wide diversity of hosts has been demonstrated in greenhouse studies [48], [64], [65], suggesting that *V. longisporum* could have originated on many different hosts. We considered one host in detail, horseradish in Illinois where both *V. longisporum* and *V. dahliae* are pathogenic and are isolated from diseased plants [62]. We found that all *V. longisporum* isolates from horseradish in Illinois belonged to *V. longisporum* lineage A1/D2. None of the three *V. dahliae* isolates (strains PD727, PD728, PD729) from the same host and location were in contention as parents of *V. longisporum*, as they grouped in the main clade of *V. dahliae*, distantly related to the *V. longisporum* parents *V. dahliae* lineages D2 and D3 (Figure 2). Similarly, all other *V. dahliae* isolates (strains PD584, PD585, PD623, PD717, PD718, PD719) from typical *V. longisporum* hosts (cabbage, oilseed rape) grouped in the main clade of *V. dahliae* (Figure 2).

Further support that *V. longisporum* originated on unusual hosts for both parents comes from *V. dahliae* strains PD327, PD362 and PD502, which are part of *V. dahliae* lineage D2, the only *V. longisporum* ancestor for which free-living, haploid strains are known (Figure 2). *Verticillium dahliae* strains PD327, PD362 and PD502 were isolated from pepper in California and maple in Wisconsin, neither of which is a host of *V. longisporum*. Pathogenicity tests of one of the isolates, *V. dahliae* strain PD327, on a variety of different hosts showed that *V. dahliae* strain PD327 was more virulent on its original host than on the *V. longisporum* hosts cabbage and cauliflower [65]. However, more research is needed to conclusively identify the sites of hybridization in the ancestry of *V. longisporum*.

### *Verticillium longisporum*, a new hybrid fungal pathogen with an expanded host range

Hybridization can lead to the evolution of new phenotypes. *Verticillium longisporum* has larger conidia than any other species of *Verticillium*, and it also is exceptionally virulent on oilseed rape in Europe and Japan [28], where it may severely reduce yields. In comparative studies of the infection process in oilseed rape, *V. longisporum* was more virulent than *V. dahliae*, one of its parental species. Both *V. longisporum* and *V. dahliae* entered the plant by penetrating the roots, but only *V. longisporum* spread to the shoot and induced disease symptoms, including stunted growth, chlorosis and discoloration of the vessels in older leaves [29]. The differences in virulence suggest a particular adaptation to oilseed rape of *V. longisporum* relative to *V. dahliae*, which possibly includes suppression of or resistance to the host defense response [29]. The differences observed between the infection process of *V. dahliae* and *V. longisporum* at the microscopic level agreed with the fact that *V. longisporum* is the most important Verticillium wilt-inducing pathogen of oilseed rape and other crucifer crops such as cabbage and cauliflower [48], suggesting that the increased virulence of *V. longisporum* might be related to its hybrid origin.

In *Phytophthora*, a group of fungal-like protists, there are examples of new plant pathogens arising through hybridization between different species [66], [67]. In fungi, a major group of plant pathogens, reports of hybrids are rare [25], and in the few known examples, the virulence and host ranges of the hybrids is generally equal or reduced relative to their parents, or unknown [4], [5], [8], [9], [10], [11], [12], [13], [14], [15], [68], [69]. The only example, to our knowledge, with increased virulence of a hybrid is the example of the poplar rust fungus *Melampsora* x *columbiana* that became the dominant species on poplar in the Pacific Northwest in 1998 by replacing one of its parents [3]. Another example for increased virulence of a fungal hybrid might be the human pathogen *Cryptococcus neoformans*, where hybrids between different lineages of the fungus occur in nature. These hybrids were artificially recreated, and were shown to have increased virulence in an animal model [16].

*Verticillium longisporum* might be one of the first examples of a hybrid fungal pathogen with a new host range. The strains of *V. longisporum* used in our study were isolated from various diseased agricultural crops, including oilseed rape where *V. longisporum* is more virulent than any of its parents, presumably due to its allodiploid status. In plants, phenotypic variation in polyploids might be due to changes in gene expression, altered regulatory interactions, and rapid genetic and epigenetic changes [2]. In fungi, little is known about the molecular consequences of polyploidy.

### Potential phenotypic differences between the *V. longisporum* hybrid lineages

There is evidence that the three *V. longisporum* lineages differ phenotypically in their host range and pathogenicity. Zeise and von Tiedemann [63] found that the isolates of their *V. longisporum* group lsp* were avirulent on oilseed rape, whereas all isolates from group lsp caused disease. We included representatives of both groups, and showed that *V. longisporum* groups lsp* and lsp corresponded to *V. longisporum* lineages A1/D3 and A1/D1, respectively. Interestingly, one parent of the oilseed rape pathogen *V. longisporum* lineage A1/D3 is *V. dahliae*, which is not pathogenic on oilseed rape [29].

Another lineage of *V. longisporum* is lineage A1/D2, only known from horseradish in Illinois [62]. Whether *V. longisporum* lineage A1/D2 causes disease on oilseed rape is not known. Considerable acreage of oilseed rape is grown in relative proximity to Illinois, in Kansas, Oklahoma, Minnesota and North Dakota (http://www.uscanola.com/, accessed on June 17, 2010), but Verticillium wilt disease on oilseed rape has never been reported from North America [28], despite the fact that no commercially grown oilseed rape varieties are resistant to *V. longisporum* [28]. This suggests that *V. longisporum* lineage A1/D2, which is confined to horseradish in Illinois, might not be pathogenic on oilseed rape. However, the agent of Verticillium wilt of oilseed rape, *V. longisporum* lineage A1/D1, is present in North America as a pathogen of cauliflower in California [48], and is pathogenic on oilseed rape in greenhouse assays [63].

### *Verticillium longisporum* originated recently

The three lineages of *V. longisporum* are genetically homogenous. Based on the seven different loci examined, there is no variation within the lineages except for one substitution in *MAT1-1* in one of the isolates (Figure 2, Figure 3). This suggests a recent origin of all three lineages, which is further supported by the fact that the alleles of species A1, a parent of all three *V. longisporum* lineages, are all identical regardless of lineage. Brasier [24] proposed that movement of fungi away from where they initially evolved might allow them to come into contact with other species, hybridize and evolve into new pathogens. We don't know whether this applies to *V. longisporum*. However, the lack of genetic variation suggests recent origin, conceivably facilitated through human activities.

The geographic locations where *V. longisporum* originated are uncertain. Lineage A1/D2 is restricted to Illinois where it might have originated; lineage A1/D3 is confined to Europe and Japan; and lineage A1/D1 occurs in Europe, Japan and North America. Population genetics studies might shed light on the centers of origins of these two lineages, as might finding all *V. longisporum* parents, which, with the exception of *V. dahliae*, are yet unknown.

### Summary and further research

Our data demonstrate that *V. longisporum* originated three different times, involving three different parental species. Further work will focus on identification and characterization of all parental species, which will provide the foundations for further research into the effects of hybridization on the parental genomes of *V. longisporum*, including the basis of pathogenicity and virulence. This work will allow for the correct identification and differentiation of the *V. longisporum* lineages which is important for disease management and enforcement of quarantine regulations.

## Materials and Methods

### Taxon sampling and origins of fungal strains

The isolates included in this study were identified based on morphological characters, and selected to represent all five species of *Verticillium* [39]. A total of 203 isolates were used, 154 isolates of *V. dahliae*, 42 isolates of *V. longisporum*, five isolates of *V. albo-atrum*, and one isolate each of *V. tricorpus* and *V. nubilum* (Table S2). The isolates were from 43 different hosts, from 16 countries and four continents.

### DNA extraction, PCR amplification for direct sequencing and DNA sequencing conditions

Fungi were grown on cellophane membranes placed on PDA plates. Mycelia were peeled off, freeze-dried and ground using liquid nitrogen. DNA was extracted with the FastDNA Kit in conjunction with the FastPrep Instrument (MP Biomedicals, Irvine, CA). Buffer CLS-Y was used, and grinding intensity was set to 4.5 for 30 seconds. PCRs were performed using GoTaq Colorless Master Mix (Promega Corp., Madison, WI, USA) in a 25 µl reaction volume according to the manufacturer's instructions. The PCR program consisted of a 2 min initial denaturation step at 94°C, 32 cycles of 10 sec at 94°C, 20 sec at the primer pair dependent annealing temperature, and 1 min at 72°C, followed by a final extension of 7 min at 72°C. PCR products were purified by sodium acetate precipitation [70]. DNA sequences were determined at the UC Davis DNA Sequencing Facility, using ABI BigDye Terminator v3.1 Cycle Sequencing chemistry on an ABI 3730 Capillary Electrophoresis Genetic Analyzer (Applied Biosystems, Foster City, CA, USA).

### Loci used for phylogenetic analyses and primer design

Eight loci were PCR amplified and sequenced with primer pairs designed based on conserved regions in the *V. dahliae* and *V. albo-atrum* genomes accessed on the BROAD Institute website (http://www.broadinstitute.org/annotation/genome/verticillium_dahliae/MultiHome.html, accessed February 10, 2009), and the *V. dahliae MAT1-1* sequence AB469828 retrieved from GenBank. Some primers were taken from the literature. The ribosomal internal transcribed spacer region was amplified with ITS1-F [71] and ITS4, and sequenced with ITS5 and ITS4 [72]. Parts of the following protein coding genes were PCR amplified and sequenced: *actin* (*ACT*) with primer pair VActF/VActR, *elongation factor 1-alpha* (*EF*) with VEFf/VEFr, *glyceraldehyde-3-phosphate dehydrogenase* (*GPD*) with primer pair VGPDf2/VGPDr, *mitochondrial oxaloacetate transport protein* (*OX*) with VOx3f/VOx2r, *tryptophan synthase* (*TS*) with VTs3f/VTs3r and *beta-tubulin* (*TUB*) with VTubF2/VTubR. Parts of the *MAT1-1* idiomorph spanning from *MAT1-1-1* to *MAT1-1-2* were amplified with Alf/MAT12r, and sequenced with various primers. For details on loci, primer sequences, PCR conditions, and sequencing primers used, see Table S4, Table S5 and Table S6.

For *V. longisporum*, allele specific primers were designed for six non-ribosomal loci (*ACT, EF, GPD, OX, TS, MAT)* based on cloned PCR products obtained with the primer pairs mentioned above. For *ACT*, the allele specific primers were ActFa1, ActF2d1, and ActF2d2, paired with reverse primer VActR; for *EF*, EFfa1, EFfd1 and EFfd2, paired with VEFr; for *GPD*, GPDfa1, GPDfd1, and GPDfd2, paired with VGPDr; for *OX*, OxFa1, OxFd1 and OxFd2 paired with VOxR; for *TS*, TsFa1, TsF2d1 and TsFd2, paired with VTs2r; and for the *MAT1-1* idiomorph, the primer pairs MATa1f/MATa1r and MATdf/MATdr. For more details on loci and PCR conditions, see Table S7.

### *MAT* screening

All isolates were screened for presence of the mating type idiomorphs *MAT1-1* and *MAT1-2* by PCR, using primers designed based on the sequenced *V. albo-atrum* and *V. dahliae* strains which were *MAT1-1* and *MAT1-2*, respectively. The primer pair Alf/MAT11r targeted parts of *MAT1-1-1*, whereas HMG21f/MAT21r targeted parts of *MAT1-2-1*. For primer sequences and PCR conditions, see Table S5 and Table S6.

### Cloning of mixed PCR products in *Verticillium longisporum*

Direct sequencing of PCR products for all but the ITS region failed in *V. longisporum*, indicating the presence of a mixed template population. To determine the number of alleles present at each locus, PCR products were cloned. To avoid formation of chimera PCR products due to the presence of mixed template, the following guidelines proposed by Beser et al. [73] were implemented. As compared to standard PCR conditions, the denaturation temperature, elongation time, and primer concentrations were increased, and the number of PCR cycles was decreased, resulting in the following PCR protocol. PCR reactant concentrations 0.8 mM for each dNTP and 0.8 µM for each primer, in a 25 µl reaction volume containing 10 or 100 ng of template DNA. To reduce PCR-induced substitutions, Easy-A High-Fidelity PCR cloning Enzyme or PfuUltra High-Fidelity DNA polymerase (Agilent Technologies, Stratagene Products Division, La Jolla, CA) were used according to the manufacturer's instruction, and the PCR program consisted of a 3 min initial denaturation at 94°C, followed by 22 cycles of 1 min at 96°C, 1 min at the primer pair dependent annealing temperature, and 4.5 min at 72°C, and followed by a final extension of 7 min at 72°C. For primer specific annealing temperatures, see Table S6. PCR products were purified with the DNA Clean & Concentrator-5 Kit (Zymo Research Corp., Orange, CA) according to the manufacturer's instructions, with an additional washing step using 80% ethanol, and eluted in a final volume of 6 µl. In case of multiple PCR bands, target bands were purified using a Zymoclean Gel DNA Recovery Kit (Zymo Research Corp., Orange, CA) following the manufacturer's instructions. PCR products were ligated to a pGEM-T Vector and cloned using the JM109 High Efficiency Competent Cells (Promega Corp., Madison, WI, USA) following the manufacturer's instructions. Transformant colonies were screened for the presence of insert by PCR with the standard M13f/M13r primers. PCR products of expected lengths were sequenced with the initial PCR primers.

### Phylogenetic analyses

Four different datasets were analyzed, including the ITS dataset, a combined five-locus dataset consisting of concatenated ITS, *ACT*, *GPD*, *EF*, *OX* and *TS* datasets, a *MAT1-1* dataset, as well as a *TUB* dataset. The *MAT1-1* outgroup sequence of *V. albo-atrum* was retrieved from the *V. albo-atrum* genome sequence from the BROAD Institute website (http://www.broadinstitute.org/annotation/genome/verticillium_dahliae/MultiHome.html, accessed February 10, 2009).

Three different algorithms were used. The ITS and *TUB* datasets were analyzed under the maximum parsimony criterion using PAUP v.4.0b 10 [74]. The combined, five-locus and the *MAT1-1* datasets were analyzed using parsimony, maximum likelihood as implemented in PAUP v.4.0b 10 [74], as well as MrBayes v3.0b4 [75] implementing a Bayesian approach to inferring phylogenies.

Most parsimonious trees were inferred using 30 random addition replicates; otherwise, default settings were used, including treating insertion/deletion gaps as missing data. Bootstrap support values were based on 500 replicates. Maximum likelihood analyses were performed using default settings and 30 random addition replicates, and bootstrap supports were based on 500 replicates. Bayesian analyses were performed with default settings, running four chains over 10 million generations and sampling each 100^th^ tree. The first 1000 of the 10,000 saved trees were discarded and the consensus tree was based on the remaining 9,000 trees. Maximum likelihood and Bayesian analyses implemented an optimal model of DNA sequence evolution determined using Modeltest 3.7 [76]. All analyses were run with a single representative of each haplotype to speed up analyses.

## Supporting Information

Figure S1 **PCR gels documenting the presence of allele A1 in all 42 isolates of** ***Verticillium longisporum*** **using allele A1 specific primers.** Loci are indicated on the left. Numbers in the top row refer to *V. longisporum* isolates as they appear in Table S2, except for strains PD356, PD402, PD629, PD730, PD589, PD614, PD687 and PD715 which are in lanes 35–42. ‘L’ indicates DNA size standards (arrowhead  =  500 bp), ‘C’ PCR negative controls. For names of loci, details on primers and PCR conditions, see text.
(TIF)

Figure S2 **PCR gels documenting the distribution of allele D1 in all 42 isolates of** ***Verticillium longisporum*** **using allele D1 specific primers.** Allele D1 was absent in *V. longisporum* strains PD356, PD402, PD629, PD730, PD589, PD614, PD687 and PD715, corresponding to lanes 35 – 42. The bands for locus *TS* in lanes 35 – 42 were due to non-specific amplification of alleles D2 or D3 by allele D1 specific primers as confirmed by DNA sequencing. Numbers in the top row refer to *V. longisporum* isolates as they appear in Table S2, except for strains PD356, PD402, PD629, PD730, PD589, PD614, PD687 and PD715 which are in lanes 35–42. ‘L’ indicates DNA size standards (arrowhead  =  500 bp), ‘C’ PCR negative controls. For names of loci, details on primers and PCR conditions, see text.
(TIF)

Figure S3 **PCR gels documenting the distribution of alleles D2 and D3 in all 42 isolates of** ***Verticillium longisporum*** **using a primer set specific to alleles D2 and D3.** Alleles D2 and D3 were absent in all but *V. longisporum* strains PD356, PD402, PD629, PD730, PD589, PD614, PD687 and PD715, corresponding to lanes 35 – 42. The bands for locus *OX* in lanes 1 – 34 were due to non-specific amplification of allele D1 as confirmed by DNA sequencing. Numbers in the top row refer to *V. longisporum* isolates as they appear in Table S2, except for strains PD356, PD402, PD629, PD730, PD589, PD614, PD687 and PD715 which are in lanes 35–42. ‘L’ indicates DNA size standards (arrowhead  =  500 bp), ‘C’ PCR negative controls. For names of loci, details on primers and PCR conditions, see text.
(TIF)

Figure S4 **PCR gels documenting the distribution of** ***MAT1-1*** **and** ***MAT1-2*** **idiomorphs in all 203** ***Verticillium*** **isolates using idiomorph specific primers.** Each of the four panes shows presence and absence of *MAT1-1* and *MAT1-2* in a subset of isolates. The order of the isolates is as in Table S2, isolate numbers are given above the panes for isolates near the DNA size standards (arrowheads  =  500 bp). ‘C’ stands for PCR negative control. Each isolate amplifies for either *MAT1-1* or *MAT1-2*, except the three isolates marked by asterisks corresponding to *V. nubilum* strain PD621, *V. tricorpus* strain PD660 and *V. dahliae* strain PD707, respectively, which failed to amplify for either primer set.
(TIF)

Figure S5 **Evolutionary origins of the diploid hybrid** ***Verticillium longisporum*** **based on phylogenetic inference from the** ***ACT*** **dataset comprising 95 taxa and 532 characters.** Shown is the single, most parsimonious tree, 199 steps in length. Isolates are represented by a strain identifier, *V. longisporum* identifiers are followed by an allele designation. Hosts and geographic origins are given. Branches with 100% bootstrap support are in bold, other support values above 70% are given by the branches.
(TIF)

Figure S6 **Evolutionary origins of the diploid hybrid** ***Verticillium longisporum*** **based on phylogenetic inference from the** ***EF*** **dataset comprising 95 taxa and 600 characters.** Shown is one most parsimonious tree, 318 steps in length. Isolates are represented by a strain identifier, *V. longisporum* identifiers are followed by an allele designation. Hosts and geographic origins are given. Branches with 100% bootstrap support are in bold, other support values above 70% are given by the branches.
(TIF)

Figure S7 **Evolutionary origins of the diploid hybrid** ***Verticillium longisporum*** **based on phylogenetic inference from the** ***GPD*** **dataset comprising 95 taxa and 678 characters.** Shown is the single, most parsimonious tree, 165 steps in length. Isolates are represented by a strain identifier, *V. longisporum* identifiers are followed by an allele designation. Hosts and geographic origins are given. Branches with 100% bootstrap support are in bold, other support values above 70% are given by the branches.
(TIF)

Figure S8 **Evolutionary origins of the diploid hybrid** ***Verticillium longisporum*** **based on phylogenetic inference from the** ***OX*** **dataset comprising 95 taxa and 606 characters.** Shown is the single, most parsimonious tree, 214 steps in length. Isolates are represented by a strain identifier, *V. longisporum* identifiers are followed by an allele designation. Hosts and geographic origins are given. Branches with 100% bootstrap support are in bold, other support values above 70% are given by the branches.
(TIF)

Figure S9 **Evolutionary origins of the diploid hybrid** ***Verticillium longisporum*** **based on phylogenetic inference from the** ***TS*** **dataset comprising 95 taxa and 591 characters.** Shown is one most parsimonious tree, 243 steps in length. Isolates are represented by a strain identifier, *V. longisporum* identifiers are followed by an allele designation. Hosts and geographic origins are given. Branches with 100% bootstrap support are in bold, other support values above 70% are given by the branches.
(TIF)

Table S1 **Numbers of PCR product clones sequenced for each** ***Verticillium longisporum*** **strain at each locus.** For details on strains, see Table S2.
(DOC)

Table S2 **Fungal isolates used; given are the strain identifiers used in this study, additional strain identifiers, the host scientific and common names, the location and date of collection, the source, the number of loci sequenced, as well as the mating type.**
(DOC)

Table S3 **Support values above 70 within** ***Verticillium dahliae*** **not given in** **Figure 2** **.**
(DOC)

Table S4 **Loci used for phylogenetic analyses; details of chromosomal locations, lengths of amplicons, introns and intergenic spacers, and locus IDs are given with respect to the sequenced genome of** ***V. dahliae*** **strain PD322 on the Broad Institute website (** http://www.broadinstitute.org/annotation/genome/verticillium_dahliae/MultiHome.html **, accessed February 10, 2009).**
(DOC)

Table S5 **The DNA sequences of all primers used are listed by primer name in ascending alphanumeric order. **Primer sequences are given 5′->3′.
(DOC)

Table S6 **PCR conditions used in this study.** For all loci, forward and reverse PCR primers, annealing temperature, annealing temperature for cloning, and expected product length in *V. dahliae* strain PD322 are given. For details on PCR conditions, see text.
(DOC)

Table S7 **Loci and conditions used for** ***V. longisporum*** **allele-specific PCR amplifications.** For each locus and allele, forward and reverse primers, annealing temperature, amplicon length with respect to *V. dahliae* strain PD322, numbers of introns targeted and total intron lengths are given. For details on PCR conditions, see text.
(DOC)
