# Supplementary figures and images for: The Ascomycete Verticillium longisporum Is a Hybrid and a Plant Pathogen with an Expanded Host Range
Source: PLoS One. 2011 Mar 24;6(3):e18260. doi: 10.1371/journal.pone.0018260 (PMC3063834; doi:10.1371/journal.pone.0018260)

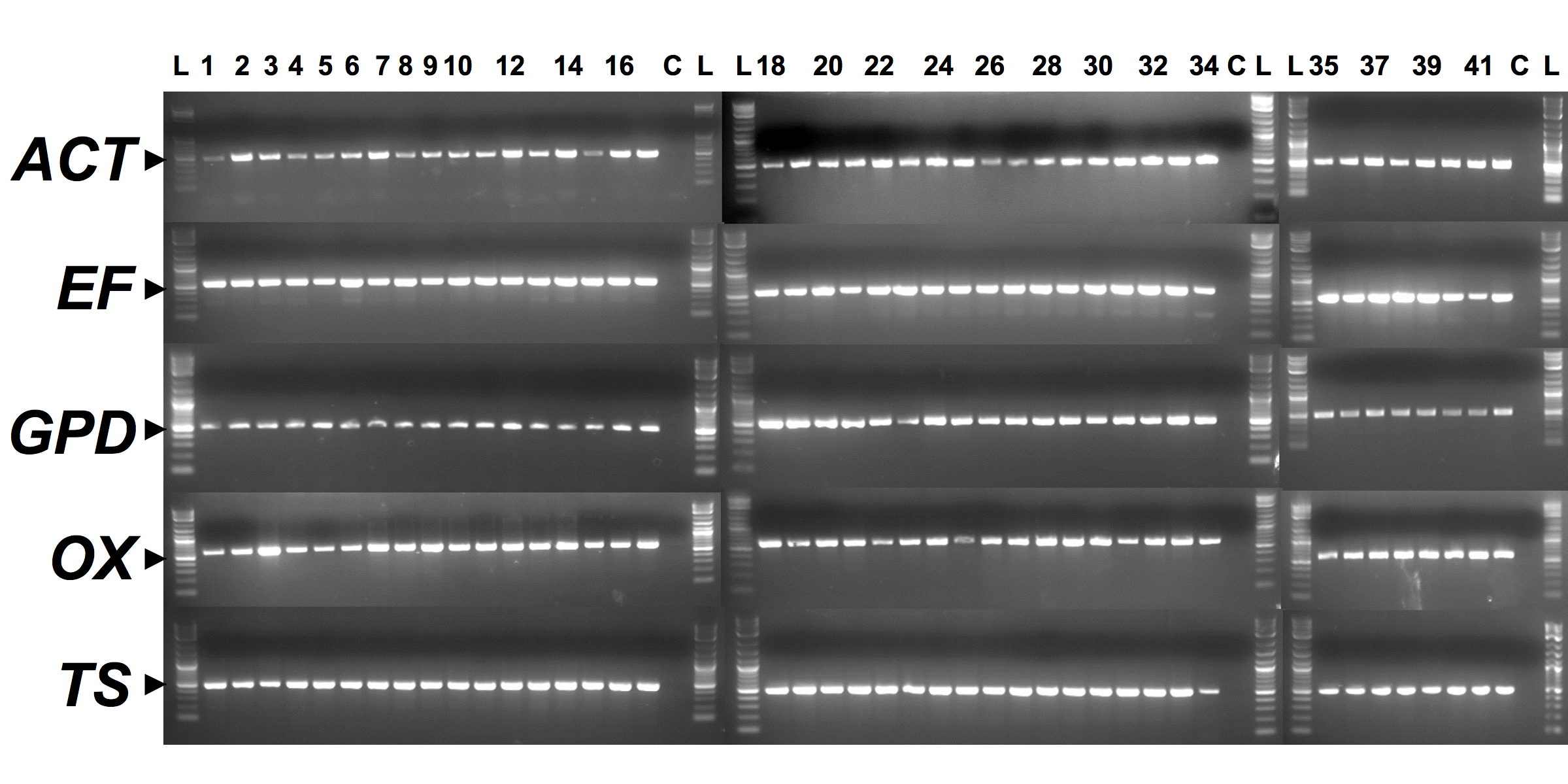

Supplement: Figure S1 — PCR gels documenting the presence of allele A1 in all 42 isolates of Verticillium longisporum using allele A1 specific primers. Loci are indicated on the left. Numbers in the top row refer to V. longisporum isolates as they appear in Table S2, except for strains PD356, PD402, PD629, PD730, PD589, PD614, PD687 and PD715 which are in lanes 35–42. ‘L’ indicates DNA size standards (arrowhead = 500 bp), ‘C’ PCR negative controls. For names of loci, details on primers and PCR conditions, see text. (TIF) [file pone.0018260.s001.tif]

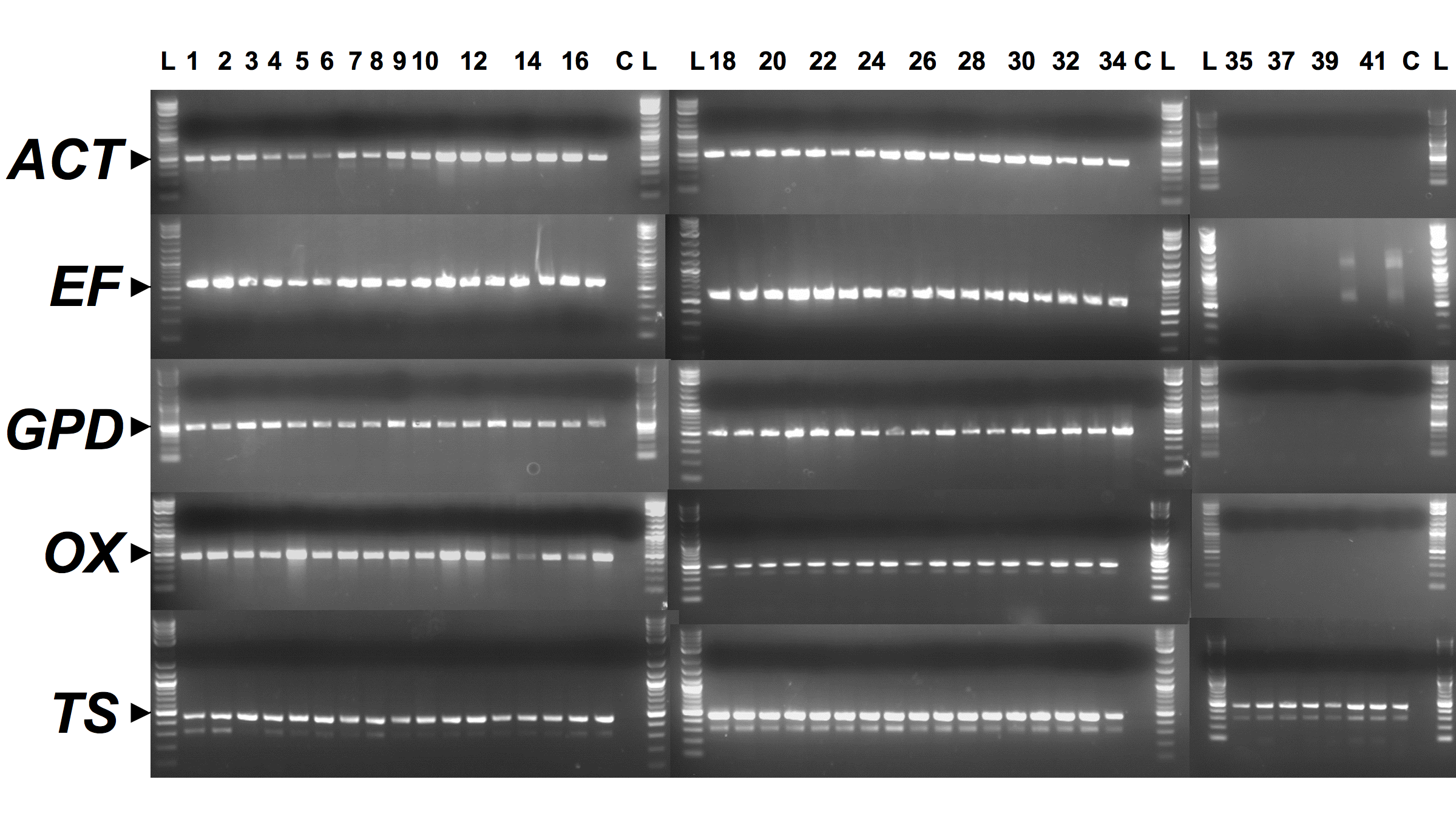

Supplement: Figure S2 — PCR gels documenting the distribution of allele D1 in all 42 isolates of Verticillium longisporum using allele D1 specific primers. Allele D1 was absent in V. longisporum strains PD356, PD402, PD629, PD730, PD589, PD614, PD687 and PD715, corresponding to lanes 35 – 42. The bands for locus TS in lanes 35 – 42 were due to non-specific amplification of alleles D2 or D3 by allele D1 specific primers as confirmed by DNA sequencing. Numbers in the top row refer to V. longisporum isolates as they appear in Table S2, except for strains PD356, PD402, PD629, PD730, PD589, PD614, PD687 and PD715 which are in lanes 35–42. ‘L’ indicates DNA size standards (arrowhead = 500 bp), ‘C’ PCR negative controls. For names of loci, details on primers and PCR conditions, see text. (TIF) [file pone.0018260.s002.tif]

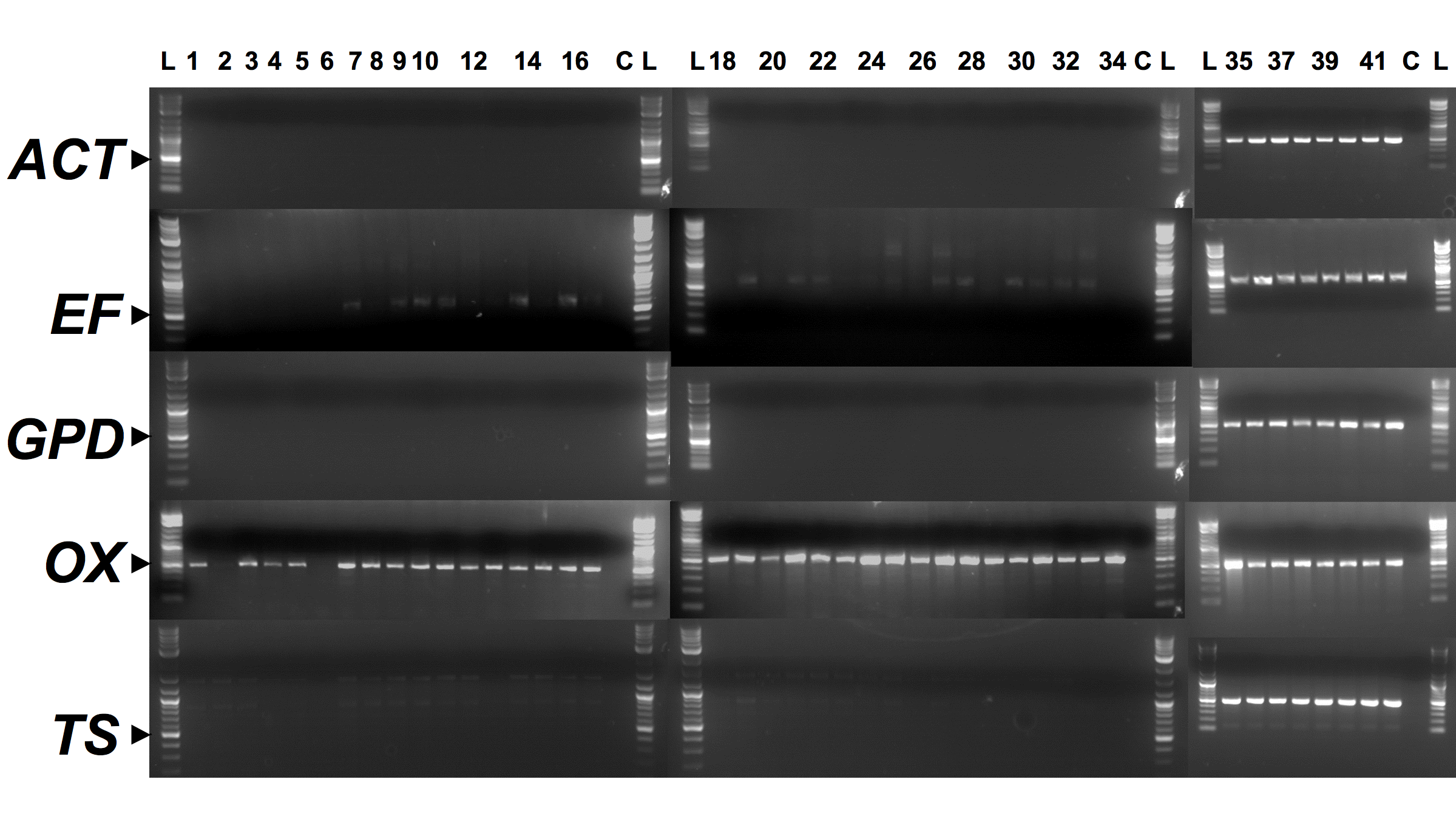

Supplement: Figure S3 — PCR gels documenting the distribution of alleles D2 and D3 in all 42 isolates of Verticillium longisporum using a primer set specific to alleles D2 and D3. Alleles D2 and D3 were absent in all but V. longisporum strains PD356, PD402, PD629, PD730, PD589, PD614, PD687 and PD715, corresponding to lanes 35 – 42. The bands for locus OX in lanes 1 – 34 were due to non-specific amplification of allele D1 as confirmed by DNA sequencing. Numbers in the top row refer to V. longisporum isolates as they appear in Table S2, except for strains PD356, PD402, PD629, PD730, PD589, PD614, PD687 and PD715 which are in lanes 35–42. ‘L’ indicates DNA size standards (arrowhead = 500 bp), ‘C’ PCR negative controls. For names of loci, details on primers and PCR conditions, see text. (TIF) [file pone.0018260.s003.tif]

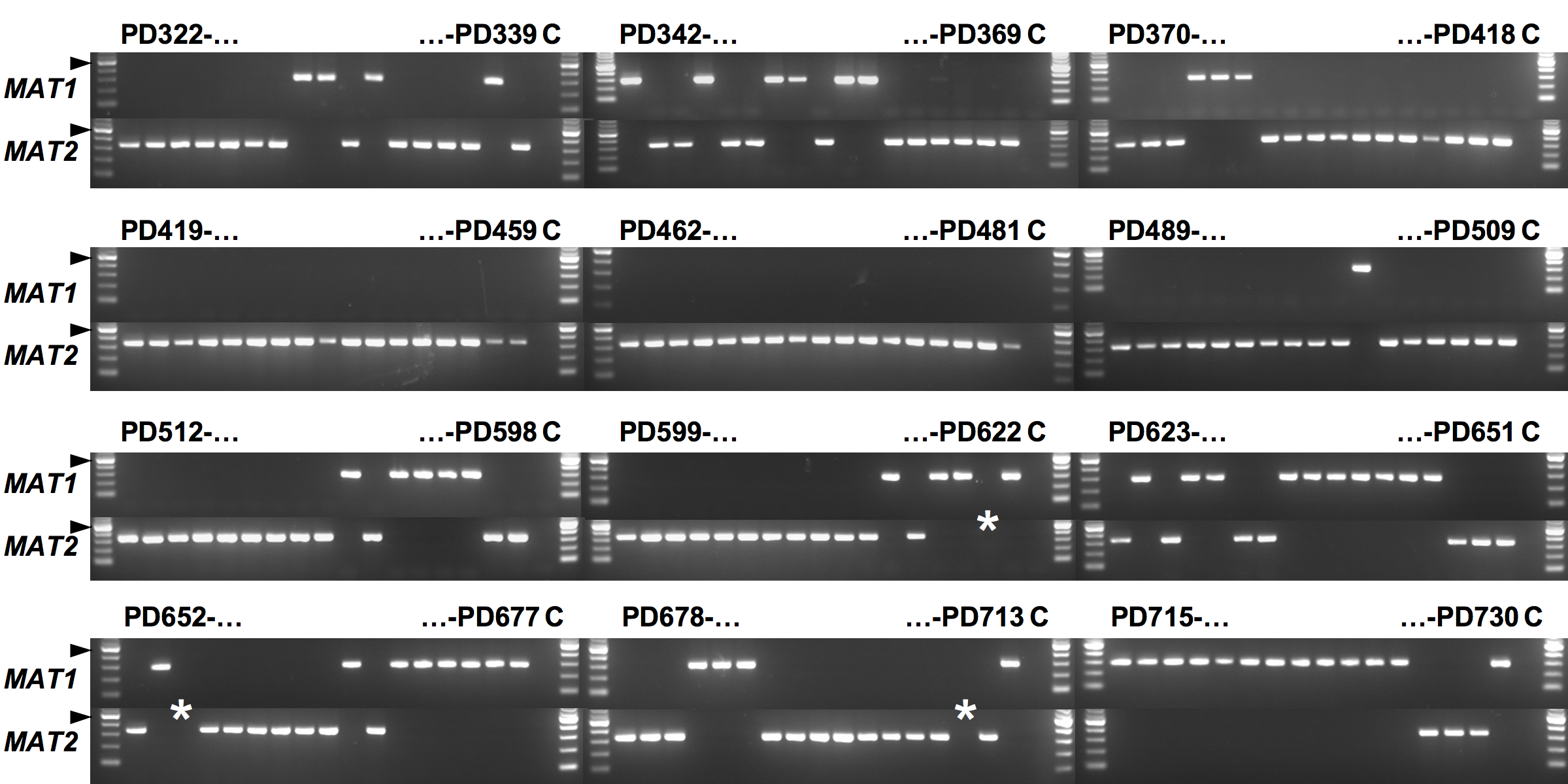

Supplement: Figure S4 — PCR gels documenting the distribution of MAT1-1 and MAT1-2 idiomorphs in all 203 Verticillium isolates using idiomorph specific primers. Each of the four panes shows presence and absence of MAT1-1 and MAT1-2 in a subset of isolates. The order of the isolates is as in Table S2, isolate numbers are given above the panes for isolates near the DNA size standards (arrowheads = 500 bp). ‘C’ stands for PCR negative control. Each isolate amplifies for either MAT1-1 or MAT1-2, except the three isolates marked by asterisks corresponding to V. nubilum strain PD621, V. tricorpus strain PD660 and V. dahliae strain PD707, respectively, which failed to amplify for either primer set. (TIF) [file pone.0018260.s004.tif]

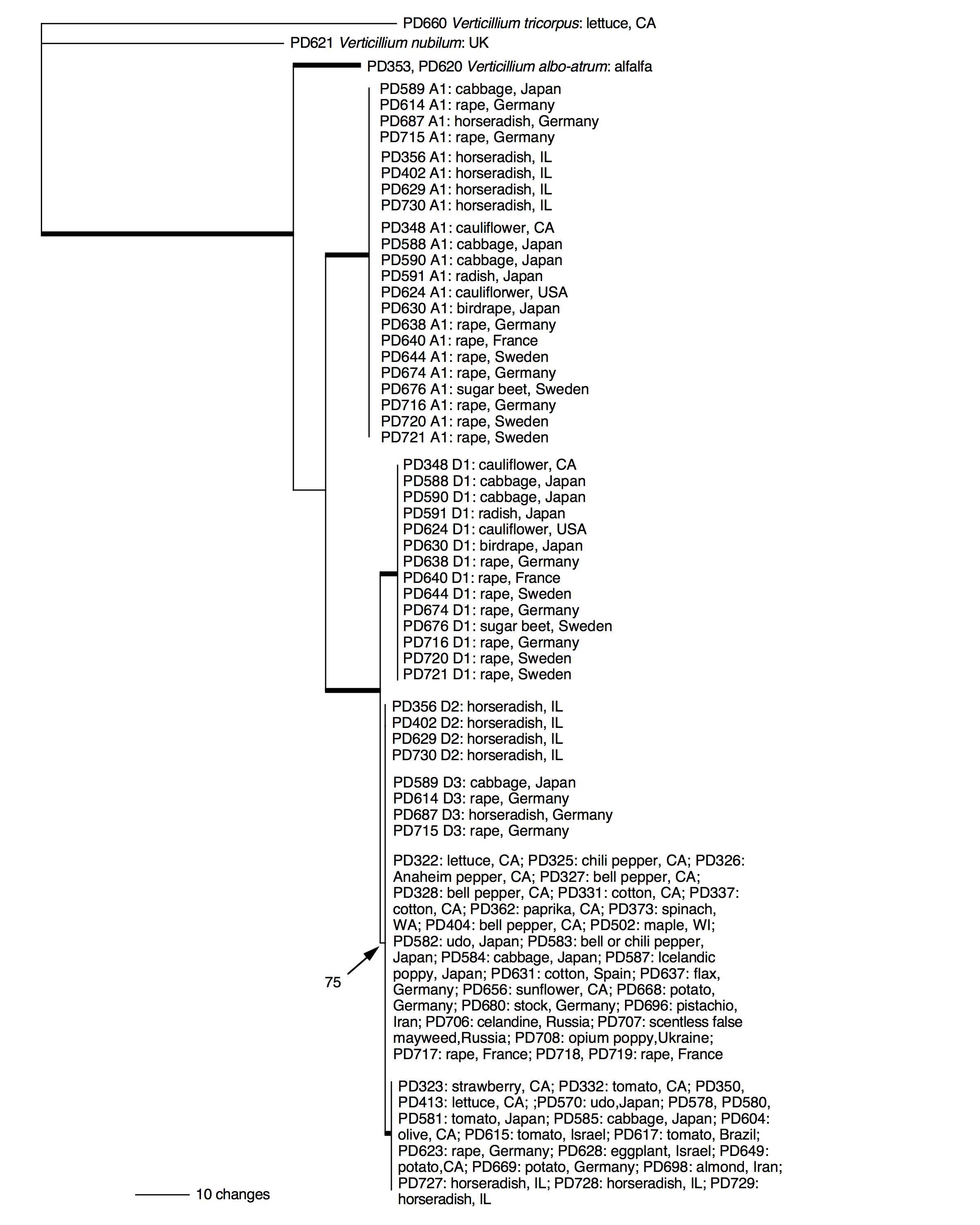

Supplement: Figure S5 — Evolutionary origins of the diploid hybrid Verticillium longisporum based on phylogenetic inference from the ACT dataset comprising 95 taxa and 532 characters. Shown is the single, most parsimonious tree, 199 steps in length. Isolates are represented by a strain identifier, V. longisporum identifiers are followed by an allele designation. Hosts and geographic origins are given. Branches with 100% bootstrap support are in bold, other support values above 70% are given by the branches. (TIF) [file pone.0018260.s005.tif]

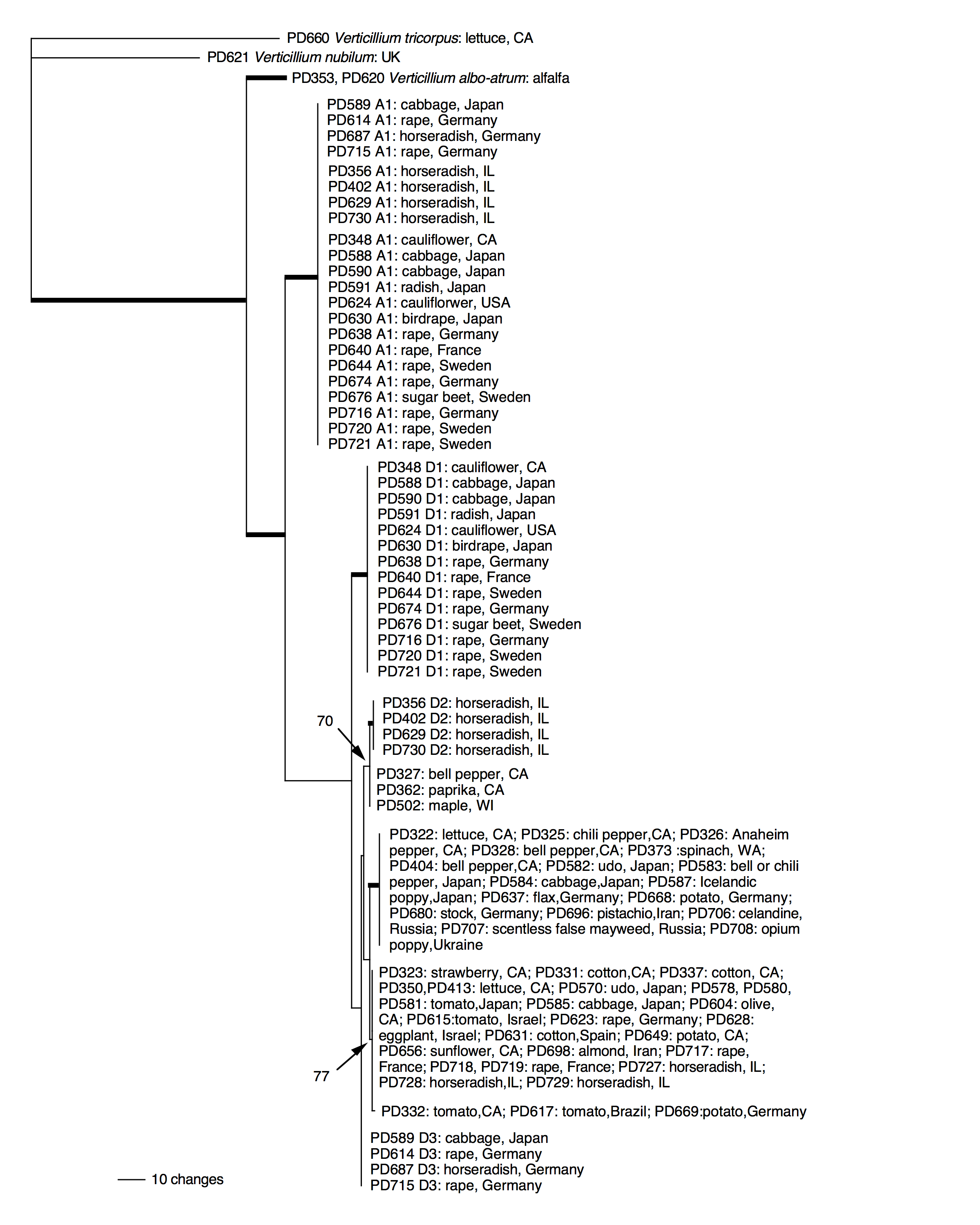

Supplement: Figure S6 — Evolutionary origins of the diploid hybrid Verticillium longisporum based on phylogenetic inference from the EF dataset comprising 95 taxa and 600 characters. Shown is one most parsimonious tree, 318 steps in length. Isolates are represented by a strain identifier, V. longisporum identifiers are followed by an allele designation. Hosts and geographic origins are given. Branches with 100% bootstrap support are in bold, other support values above 70% are given by the branches. (TIF) [file pone.0018260.s006.tif]

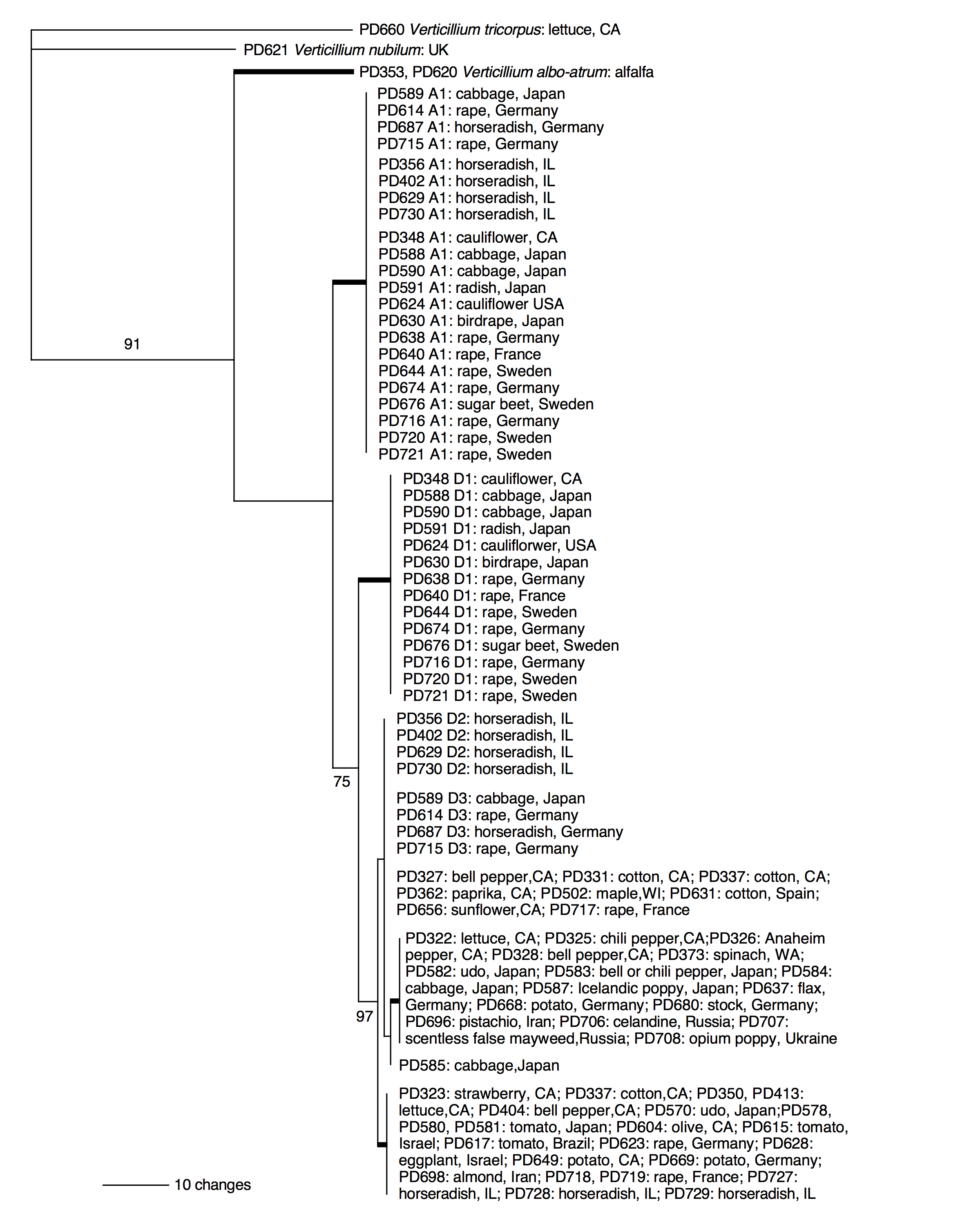

Supplement: Figure S7 — Evolutionary origins of the diploid hybrid Verticillium longisporum based on phylogenetic inference from the GPD dataset comprising 95 taxa and 678 characters. Shown is the single, most parsimonious tree, 165 steps in length. Isolates are represented by a strain identifier, V. longisporum identifiers are followed by an allele designation. Hosts and geographic origins are given. Branches with 100% bootstrap support are in bold, other support values above 70% are given by the branches. (TIF) [file pone.0018260.s007.tif]

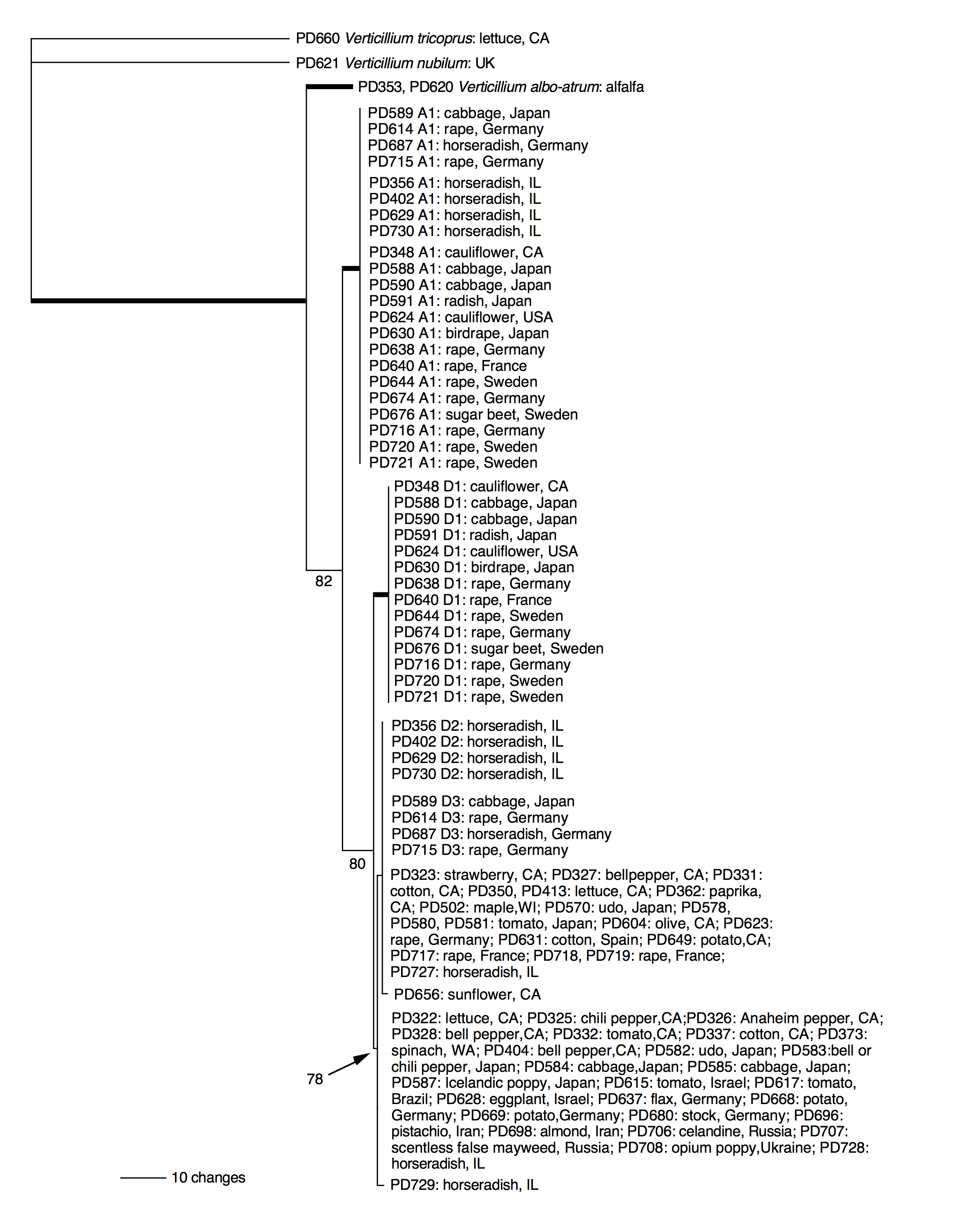

Supplement: Figure S8 — Evolutionary origins of the diploid hybrid Verticillium longisporum based on phylogenetic inference from the OX dataset comprising 95 taxa and 606 characters. Shown is the single, most parsimonious tree, 214 steps in length. Isolates are represented by a strain identifier, V. longisporum identifiers are followed by an allele designation. Hosts and geographic origins are given. Branches with 100% bootstrap support are in bold, other support values above 70% are given by the branches. (TIF) [file pone.0018260.s008.tif]

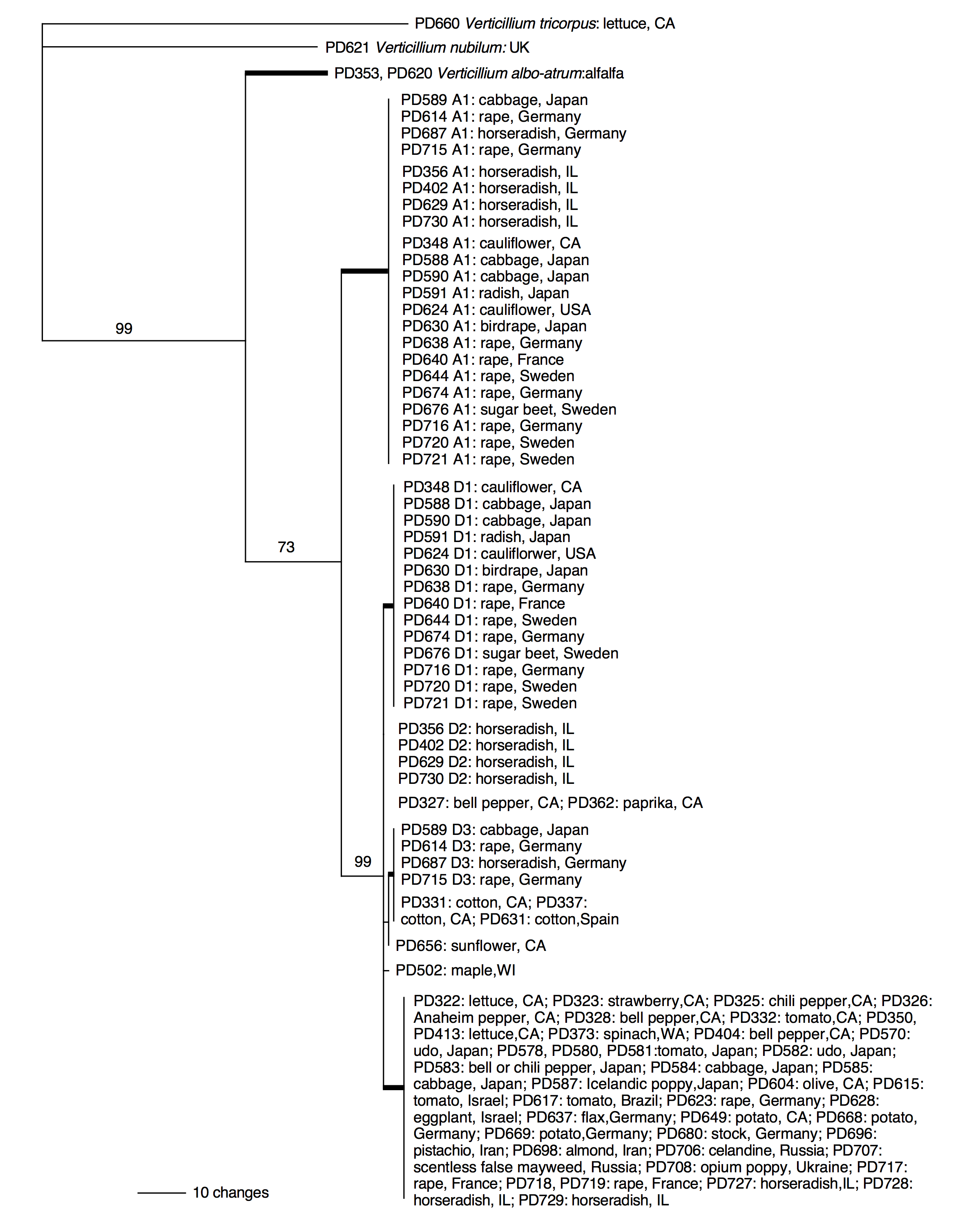

Supplement: Figure S9 — Evolutionary origins of the diploid hybrid Verticillium longisporum based on phylogenetic inference from the TS dataset comprising 95 taxa and 591 characters. Shown is one most parsimonious tree, 243 steps in length. Isolates are represented by a strain identifier, V. longisporum identifiers are followed by an allele designation. Hosts and geographic origins are given. Branches with 100% bootstrap support are in bold, other support values above 70% are given by the branches. (TIF) [file pone.0018260.s009.tif]
