# Supplementary material for: The Ascomycete Verticillium longisporum Is a Hybrid and a Plant Pathogen with an Expanded Host Range
Source: PLoS One. 2011 Mar 24;6(3):e18260. doi: 10.1371/journal.pone.0018260 (PMC3063834; doi:10.1371/journal.pone.0018260)
Supplement: Table S1 — Numbers of PCR product clones sequenced for each Verticillium longisporum strain at each locus. For details on strains, see Table S2. (DOC) [file pone.0018260.s010.doc]

| Strain | *Act* | *EF* | *GPD* | *OX* | *TS* | *MAT* | ITS | *TUB* | Totals for each isolate |
| --- | --- | --- | --- | --- | --- | --- | --- | --- | --- |
| PD322 | 0 | 0 | 0 | 0 | 0 | 0 | 0 | 4 | 4 |
| PD329 | 2 | 8 | 4 | 0 | 0 | 0 | 4 | 2 | 20 |
| PD342 | 6 | 7 | 4 | 0 | 0 | 4 | 4 | 0 | 25 |
| PD348 | 10 | 5 | 3 | 0 | 0 | 4 | 0 | 8 | 30 |
| PD356 | 4 | 5 | 4 | 0 | 0 | 4 | 0 | 4 | 21 |
| PD363 | 0 | 0 | 0 | 0 | 0 | 0 | 0 | 4 | 4 |
| PD402 | 4 | 9 | 3 | 4 | 4 | 0 | 0 | 8 | 32 |
| PD588 | 4 | 7 | 8 | 4 | 4 | 0 | 0 | 0 | 27 |
| PD591 | 4 | 3 | 7 | 0 | 0 | 0 | 0 | 0 | 14 |
| PD614 | 4 | 4 | 5 | 0 | 0 | 0 | 0 | 8 | 21 |
| PD622 | 7 | 4 | 6 | 0 | 0 | 0 | 0 | 0 | 17 |
| PD629 | 4 | 0 | 6 | 0 | 0 | 0 | 0 | 0 | 10 |
| Totals each locus/Grand total | 49 | 52 | 50 | 8 | 8 | 12 | 8 | 38 | 225 |
