# Supplementary material for: The Ascomycete Verticillium longisporum Is a Hybrid and a Plant Pathogen with an Expanded Host Range
Source: PLoS One. 2011 Mar 24;6(3):e18260. doi: 10.1371/journal.pone.0018260 (PMC3063834; doi:10.1371/journal.pone.0018260)
Supplement: Table S2 — Fungal isolates used; given are the strain identifiers used in this study, additional strain identifiers, the host scientific and common names, the location and date of collection, the source, the number of loci sequenced, as well as the mating type. (DOC) [file pone.0018260.s011.doc]

| Strain identifiers | Other Strain Identifiers | *Verticillium* species* | Host | Host common name† | Location | Collection date | Source | Loci | *MAT* |
| --- | --- | --- | --- | --- | --- | --- | --- | --- | --- |
| PD322 | Ls.17 | *V. dahliae* Kleb. | *Lactuca sativa* L. | lettuce | Watsonville, CA, USA | 1996 | Subbarao Lab Collection | 6 | *MAT1-2* |
| PD323 | Fca.23 | *V. dahliae* | *Fragaria X ananassa* Duchesne | hybrid strawberry | Watsonville, CA, USA | 1996 | Subbarao Lab Collection | 6 | *MAT1-2* |
| PD324 | Fca.29, V1 | *V. dahliae* | *Fragaria X ananassa* | hybrid strawberry | Watsonville, CA, USA | 1996 | Subbarao Lab Collection | 1 | *MAT1-2* |
| PD325 | Cf.31 | *V. dahliae* | *Capsicum annuum* L. | chili pepper‡ | Gilroy CA, USA | 1991 | Steve Koike | 6 | *MAT1-2* |
| PD326 | Cf.40 | *V. dahliae* | *Capsicum annuum* | Anaheim pepper‡ | Gilroy CA, USA | 1996 | Subbarao Lab Collection | 6 | *MAT1-2* |
| PD327 | Ca.59, B, T2 | *V. dahliae* | *Capsicum annuum* | bell pepper‡ | Gilroy CA, USA | 1996 | Subbarao Lab Collection | 6 | *MAT1-2* |
| PD328 | Ca.63 | *V. dahliae* | *Capsicum annuum* | bell pepper‡ | California, USA | 1996 | Subbarao Lab Collection | 6 | *MAT1-2* |
| PD329 | Bob.71 | *V. longisporum* (C. Stark) Karapapa, Bainbr. & Heale D1 | *Brassica oleracea var. botrytis* L. | cauliflower‡ | California, USA | 1990 | Subbarao Lab Collection | 1 | *MAT1-1* |
| PD330 | Boc.74, 91-05 | *V. longisporum* D1 | *Brassica oleracea* L. | cabbage | California, USA | 1991 | Subbarao Lab Collection | 1 | *MAT1-1* |
| PD331 | Gh.77 | *V. dahliae* | *Gossypium hirsutum* L. | upland cotton | California, USA | - | Subbarao Lab Collection | 6 | *MAT1-2* |
| PD332 | Le.78 | *V. dahliae* | *Solanum lycopersicum var. lycopersicum* L. | garden tomato | Davis, CA, USA | - | Subbarao Lab Collection | 6 | *MAT1-1* |
| PD333 | Cs.80, 91-04 | *V. dahliae* | *Cynara scolymus* L. | globe artichoke | CA, USA | 1991 | Subbarao Lab Collection | 1 | *MAT1-2* |
| PD335 | Mp.89, OC-96-3 | *V. dahliae* | *Mentha X piperita* L. | peppermint | Oregon, USA | - | Ken Johnson | 1 | *MAT1-2* |
| PD336 | St.91, O28 | *V. dahliae* | *Solanum tuberosum* L. | Irish potato | Oregon, USA | 1996 | M. Powelson | 1 | *MAT1-2* |
| PD337 | Gh.101, MD-05 | *V. dahliae* | *Gossypium hirsutum* | upland cotton | California, USA | - | Subbarao Lab Collection | 6 | *MAT1-2* |
| PD338 | Ms.107 | *V. albo-atrum* Reinke & Berthold | *Medicago sativa* L. | alfalfa | Rock Springs, PA, USA | 1986 | B. W. Pennypacker | 1 | *MAT1-1* |
| PD339 | Sm.113 | *V. dahliae* | *Solanum melongena* L. | eggplant | California, USA | 1997 | Deborah Fravel | 1 | *MAT1-2* |
| PD342 | Bno.188 | *V. longisporum* D1 | *Brassica napus* L. | rape | Germany | 1989 | K. Zeise | 1 | *MAT1-1* |
| PD344 | Ls.331 | *V. dahliae* | *Lactuca sativa* | lettuce | Salinas, CA, USA | 2000 | Subbarao Lab Collection | 1 | *MAT1-2* |
| PD346 | Ls.446 | *V. dahliae* | *Lactuca sativa* | lettuce | Salinas, CA, USA | 2001 | Subbarao Lab Collection | 1 | *MAT1-2* |
| PD348 | Bob.70, 90-02 | *V. longisporum* d1 | *Brassica oleracea var. botrytis* | cauliflower‡ | Salinas, CA, USA | 1990 | Subbarao Lab Collection | 6 | *MAT1-1* |
| PD349 | Ls.7, L-7 | *V. dahliae* | *Lactuca sativa* | lettuce | Watsonville, CA, USA | 1995 | Subbarao Lab Collection | 1 | *MAT1-2* |
| PD350 | Ls.18 | *V. dahliae* | *Lactuca sativa* | lettuce | Watsonville, CA, USA | 1996 | Subbarao Lab Collection | 6 | *MAT1-2* |
| PD352 | Bob.69 | *V. longisporum* D1 | *Brassica oleracea var. botrytis* | cauliflower‡ | Salinas, CA, USA | 1990 | Subbarao Lab Collection | 1 | *MAT1-1* |
| PD353 | Ms.102, VP-1 | *V. albo-atrum* | *Medicago sativa* | alfalfa | Smullton, PA, USA | 1986 | B. W. Pennypacker | 6 | *MAT1-1* |
| PD354 | Le.112 | *V. dahliae* | *Solanum lycopersicum var. lycopersicum* | garden tomato | California, USA | 1997 | Dennis Lawn | 1 | *MAT1-2* |
| PD355 | Bob.127 | *V. longisporum* D1 | *Brassica oleracea var. botrytis* | cauliflower‡ | California, USA | 1997 | Subbarao Lab Collection | 1 | *MAT1-1* |
| PD356 | Ar.139 | *V. longisporum* d2 | *Armoracia rusticana* P.G. Gaertn., B. Mey. & Scherb. | horseradish | Illinois, USA | 1997 | Subbarao Lab Collection | 6 | *MAT1-1* |
| PD358 | Ls.237 | *V. dahliae* | *Lactuca sativa* | lettuce | Salinas, CA, USA | 1999 | Subbarao Lab Collection | 1 | *MAT1-2* |
| PD359 | Ls.240 | *V. dahliae* | *Lactuca sativa* | lettuce | Salinas, CA, USA | 1999 | Subbarao Lab Collection | 1 | *MAT1-2* |
| PD361 | Fca.246 | *V. dahliae* | *Fragaria X ananassa* | hybrid strawberry | Salinas, CA, USA | 1999 | Subbarao Lab Collection | 1 | *MAT1-2* |
| PD362 | Ca.271 | *V. dahliae* | *Capsicum annuum* | paprika‡ | Gilroy CA, USA | 1999 | Subbarao Lab Collection | 6 | *MAT1-2* |
| PD363 | Ca.276 | *V. dahliae* | *Capsicum annuum* | jalapeno‡ | Gilroy CA, USA | 1999 | Subbarao Lab Collection | 1 | *MAT1-2* |
| PD369 | Sm.514 | *V. dahliae* | *Solanum melongena* | eggplant | Oregon, USA | 2002 | Melodie Putnum | 1 | *MAT1-2* |
| PD370 | Ep.515 | *V. dahliae* | *Echinacea purpurea* (L.) Moench | purple coneflower | Oregon, USA | 2002 | Melodie Putnum | 1 | *MAT1-2* |
| PD371 | Cc.517 | *V. dahliae* | *Cotinus coggygria* Scop. | European smoketree | Oregon, USA | 2002 | Melodie Putnum | 1 | *MAT1-2* |
| PD373 | So.912 | *V. dahliae* | *Spinacia oleracea* L. | spinach | Washington State, USA | 2001 | Lindsey du Toit | 6 | *MAT1-2* |
| PD402 | Ar.136 | *V. longisporum* d2 | *Armoracia rusticana* | horseradish | Illinois, USA | 1997 | Subbarao Lab Collection | 6 | *MAT1-1* |
| PD403 | Ca.146 | *V. dahliae* | *Capsicum annuum* | bell pepper‡ | Santa Clara County, CA, USA | 1997 | Subbarao Lab Collection | 1 | *MAT1-1* |
| PD404 | Ca.148 | *V. dahliae* | *Capsicum annuum* | bell pepper‡ | Santa Clara County, CA, USA | 1997 | Subbarao Lab Collection | 6 | *MAT1-1* |
| PD405 | Ls.435 | *V. dahliae* | *Lactuca sativa* | lettuce | Watsonville, CA, USA | 2001 | Subbarao Lab Collection | 1 | *MAT1-2* |
| PD406 | Ls.483 | *V. dahliae* | *Lactuca sativa* | lettuce | Salinas, CA, USA | 2001 | Subbarao Lab Collection | 1 | *MAT1-2* |
| PD408 | So.921 | *V. dahliae* | *Spinacia oleracea* L. | spinach | Denmark | 2003 | Lindsey du Toit | 1 | *MAT1-2* |
| PD409 | So.934 | *V. dahliae* | *Spinacia oleracea* L. | spinach | Denmark | 2003 | Lindsey du Toit | 1 | *MAT1-2* |
| PD410 | V158 | *V. dahliae* | *Solanum lycopersicum var. lycopersicum* | garden tomato | California, USA | 2001 | Subbarao Lab Collection | 1 | *MAT1-2* |
| PD411 | V163 | *V. dahliae* | *Solanum lycopersicum var. lycopersicum* | garden tomato | California, USA | 2005 | Subbarao Lab Collection | 1 | *MAT1-2* |
| PD413 | Ls.16 | *V. dahliae* | *Lactuca sativa* | lettuce | Watsonville, CA, USA | 1996 | Subbarao Lab Collection | 6 | *MAT1-2* |
| PD414 | Ca.35 | *V. dahliae* | *Capsicum annuum* | Anaheim pepper‡ | Gilroy CA, USA | 1996 | Subbarao Lab Collection | 1 | *MAT1-2* |
| PD415 | Cf.38 | *V. dahliae* | *Capsicum annuum* | Anaheim pepper‡ | King City, CA, USA | 1996 | Subbarao Lab Collection | 1 | *MAT1-2* |
| PD416 | Cf.45 | *V. dahliae* | *Capsicum annuum* | Anaheim pepper‡ | Gilroy CA, USA | 1996 | Steve Koike | 1 | *MAT1-2* |
| PD418 | Cf.56 | *V. dahliae* | *Capsicum annuum* | Anaheim pepper‡ | Gilroy CA, USA | 1996 | Subbarao Lab Collection | 1 | *MAT1-2* |
| PD419 | Ca.66 | *V. dahliae* | *Capsicum annuum* | bell pepper‡ | California, USA | 1996 | Subbarao Lab Collection | 1 | *MAT1-2* |
| PD421 | Cv.111 | *V. dahliae* | *Citrullus lanatus var. lanatus* (Thunb.) Matsumura & Nakai | watermelon | California, USA | 1993 | Dennis Lawn | 1 | *MAT1-2* |
| PD422 | Fca.414 | *V. dahliae* | *Fragaria X ananassa* | hybrid strawberry | Watsonville, CA, USA | 2000 | Subbarao Lab Collection | 1 | *MAT1-2* |
| PD424 | Cs.423 | *V. dahliae* | *Cynara scolymus* | globe artichoke | Salinas, CA, USA | 2001 | Subbarao Lab Collection | 1 | *MAT1-2* |
| PD425 | Ca.306 | *V. dahliae* | *Capsicum annuum* | bell pepper‡ | Greenfiled, CA, USA | 1999 | Subbarao Lab Collection | 1 | *MAT1-2* |
| PD426 | Ca.310 | *V. dahliae* | *Capsicum annuum* | bell pepper‡ | Greenfiled, CA, USA | 1999 | Subbarao Lab Collection | 1 | *MAT1-2* |
| PD427 | Cs.312 | *V. dahliae* | *Cynara scolymus* | globe artichoke | California, USA | 1999 | Subbarao Lab Collection | 1 | *MAT1-2* |
| PD428 | Ls.316 | *V. dahliae* | *Lactuca sativa* | lettuce | Salinas, CA, USA | 2000 | Subbarao Lab Collection | 1 | *MAT1-2* |
| PD429 | Cf.158 | *V. dahliae* | *Capsicum annuum* | chili pepper‡ | California, USA | 1997 | Subbarao Lab Collection | 1 | *MAT1-2* |
| PD430 | Cf.162 | *V. dahliae* | *Capsicum annuum* | chili pepper‡ | California, USA | 1997 | Subbarao Lab Collection | 1 | *MAT1-2* |
| PD431 | Fca.254 | *V. dahliae* | *Fragaria X ananassa* | hybrid strawberry | Salinas, CA, USA | 1999 | Subbarao Lab Collection | 1 | *MAT1-2* |
| PD445 | Ls.903 | *V. dahliae* | *Lactuca sativa* | lettuce | Watsonville, CA, USA | 2006 | Subbarao Lab Collection | 1 | *MAT1-2* |
| PD452 | Cs.455 | *V. dahliae* | *Cynara scolymus* | globe artichoke | Castroville, CA, USA | 2001 | Subbarao Lab Collection | 1 | *MAT1-2* |
| PD453 | Ca.465 | *V. dahliae* | *Capsicum annuum* | pepper‡ | California, USA | 2001 | Subbarao Lab Collection | 1 | *MAT1-2* |
| PD456 | Ca.496 | *V. dahliae* | *Capsicum annuum* | pepper‡ | Salinas, CA, USA | 2001 | Steve Koike | 1 | *MAT1-2* |
| PD457 | Ls.636 | *V. dahliae* | *Lactuca sativa* | lettuce | Watsonville, CA, USA | 2004 | Subbarao Lab Collection | 1 | *MAT1-2* |
| PD459 | Ls.711 | *V. dahliae* | *Lactuca sativa* | lettuce | Watsonville, CA, USA | 2005 | Subbarao Lab Collection | 1 | *MAT1-2* |
| PD462 | Cv.863 | *V. dahliae* | *Citrullus lanatus var. lanatus* | watermelon | Texas, USA | 2004 | Subbarao Lab Collection | 1 | *MAT1-2* |
| PD463 | Ls.851 | *V. dahliae* | *Lactuca sativa* | lettuce | Salinas, CA, USA | 2006 | Subbarao Lab Collection | 1 | *MAT1-2* |
| PD465 | Ls.797 | *V. dahliae* | *Lactuca sativa* | lettuce | Watsonville, CA, USA | 2005 | Subbarao Lab Collection | 1 | *MAT1-2* |
| PD466 | Ls.791 | *V. dahliae* | *Lactuca sativa* | lettuce | Watsonville, CA, USA | 2005 | Subbarao Lab Collection | 1 | *MAT1-2* |
| PD468 | So.919 | *V. dahliae* | *Spinacia oleracea* L. | spinach | Washington State, USA | 2003 | Lindsey du Toit | 1 | *MAT1-2* |
| PD469 | So.922 | *V. dahliae* | *Spinacia oleracea* L. | spinach | Denmark | 2003 | Lindsey du Toit | 1 | *MAT1-2* |
| PD470 | So.923 | *V. dahliae* | *Spinacia oleracea* L. | spinach | Denmark | 2003 | Lindsey du Toit | 1 | *MAT1-2* |
| PD471 | So.924 | *V. dahliae* | *Spinacia oleracea* L. | spinach | Netherlands | 2003 | Lindsey du Toit | 1 | *MAT1-2* |
| PD472 | So.925 | *V. dahliae* | *Spinacia oleracea* L. | spinach | Netherlands | 2003 | Lindsey du Toit | 1 | *MAT1-2* |
| PD474 | So.927 | *V. dahliae* | *Spinacia oleracea* L. | spinach | Netherlands | 2003 | Lindsey du Toit | 1 | *MAT1-2* |
| PD475 | So.928 | *V. dahliae* | *Spinacia oleracea* L. | spinach | Denmark | 2003 | Lindsey du Toit | 1 | *MAT1-2* |
| PD476 | So.930 | *V. dahliae* | *Spinacia oleracea* L. | spinach | Denmark | 1993 | Lindsey du Toit | 1 | *MAT1-2* |
| PD477 | So.937 | *V. dahliae* | *Spinacia oleracea* L. | spinach | Denmark | 2003 | Lindsey du Toit | 1 | *MAT1-2* |
| PD478 | So.943 | *V. dahliae* | *Spinacia oleracea* L. | spinach | Netherlands | 2003 | Lindsey du Toit | 1 | *MAT1-2* |
| PD479 | Ls.950 | *V. dahliae* | *Lactuca sativa* | lettuce | King City, CA, USA | 2006 | Steve Koike | 1 | *MAT1-2* |
| PD480 | So.953 | *V. dahliae* | *Spinacia oleracea* L. | spinach | Washington State, USA | 2006 | Steve Koike | 1 | *MAT1-2* |
| PD481 | So.965 | *V. dahliae* | *Spinacia oleracea* L. | spinach | - | - | Subbarao Lab Collection | 1 | *MAT1-2* |
| PD487 | VE0021d | *V. dahliae* | *Fragaria X ananassa* | hybrid strawberry | Watsonville, CA, USA | - | Tom Gordon | 1 | *MAT1-2* |
| PD488 | F006 | *V. dahliae* | *Solanum lycopersicum var. lycopersicum* | garden tomato | - | 1998 | Tom Gordon | 1 | *MAT1-2* |
| PD490 | Cv.85, watermelon | *V. dahliae* | *Citrullus lanatus var. lanatus* | watermelon | California, USA | 1994 | Subbarao Lab Collection | 1 | *MAT1-2* |
| PD491 | Vd9602 | *V. dahliae* | *Fragaria X ananassa* | hybrid strawberry | California, USA | - | Tom Gordon | 1 | *MAT1-2* |
| PD493 | T5AS-5 | *V. dahliae* | *Solanum lycopersicum var. lycopersicum* | garden tomato | California, USA | 1999 | Tom Gordon | 1 | *MAT1-2* |
| PD494 | Vd9602 | *V. dahliae* | *Fragaria X ananassa* | hybrid strawberry | Siskiyou County, CA, USA | 1996 | Tom Gordon | 1 | *MAT1-2* |
| PD495 | VE0021d | *V. dahliae* | *Fragaria X ananassa* | hybrid strawberry | California, USA | 2003 | Tom Gordon | 1 | *MAT1-2* |
| PD496 | VE0002i | *V. dahliae* | *Fragaria X ananassa* | hybrid strawberry | Irvine, CA, USA | prior to 2003 | Tom Gordon, John Duniway | 1 | *MAT1-2* |
| PD497 | Ls.1 | *V. dahliae* | *Lactuca sativa* | lettuce | Watsonville, CA, USA | 1995 | Subbarao Lab Collection | 1 | *MAT1-2* |
| PD500 | Fca.229 | *V. dahliae* | *Fragaria X ananassa* | hybrid strawberry | Salinas, CA, USA | 1998 | Subbarao Lab Collection | 1 | *MAT1-2* |
| PD502 | Acer.875 | *V. dahliae* | *Acer sp*. | maple | Greenfiled, WI, USA | 2006 | Glen Stanosz | 6 | *MAT1-1* |
| PD503 | Fraxinus.881 | *V. dahliae* | *Fraxinus sp.* | ash | Beloit, WI, USA | 2006 | Glen Stanosz | 1 | *MAT1-2* |
| PD504 | wi.884 | *V. dahliae* | *Fraxinus sp.* | ash | Nashoteh, WI, USA | 2006 | Glen Stanosz | 1 | *MAT1-2* |
| PD505 | Ap.889 | *V. dahliae* | *Acer palmatum* Thunb. | Japanese maple | Madison, WI, USA | 2006 | Glen Stanosz | 1 | *MAT1-2* |
| PD506 | So.910 | *V. dahliae* | *Spinacia oleracea* L. | spinach | Watsonville, CA, USA | 2006 | Subbarao Lab Collection | 1 | *MAT1-2* |
| PD508 | So.932 | *V. dahliae* | *Spinacia oleracea* L. | spinach | Denmark | 2003 | Lindsey du Toit | 1 | *MAT1-2* |
| PD509 | So.940 | *V. dahliae* | *Spinacia oleracea* L. | spinach | Netherlands | 2003 | Lindsey du Toit | 1 | *MAT1-2* |
| PD512 | Le.1092 | *V. dahliae* | *Solanum lycopersicum var. lycopersicum* | garden tomato | - | - | Subbarao Lab Collection | 1 | *MAT1-2* |
| PD570 | U22 | *V. dahliae* | *Aralia cordata* Thunb. | udo | Gunma, Japan | - | Toshiyuki Usami, Toshimasa Shiraishi | 6 | *MAT1-2* |
| PD578 | TO-2 | *V. dahliae* | *Solanum lycopersicum var. lycopersicum* | garden tomato | Gunma, Japan | - | Toshiyuki Usami, Toshimasa Shiraishi | 6 | *MAT1-2* |
| PD579 | TO-21 | *V. dahliae* | *Solanum lycopersicum var. lycopersicum* | garden tomato | Gunma, Japan | - | Toshiyuki Usami, Toshimasa Shiraishi | 1 | *MAT1-2* |
| PD580 | TK15 | *V. dahliae* | *Solanum lycopersicum var. lycopersicum* | garden tomato | Kanagawa, Japan | - | Toshiyuki Usami | 6 | *MAT1-2* |
| PD581 | TK23 | *V. dahliae* | *Solanum lycopersicum var. lycopersicum* | garden tomato | Kanagawa, Japan | - | Toshiyuki Usami | 6 | *MAT1-2* |
| PD582 | U48 | *V. dahliae* | *Aralia cordata* | udo | Gunma, Japan | - | Toshiyuki Usami, Toshimasa Shiraishi | 6 | *MAT1-2* |
| PD583 | P9-1 | *V. dahliae* | *Capsicum annuum* | bell or chili pepper‡ | Hokkaido, Japan | - | Toshiyuki Usami | 6 | *MAT1-2* |
| PD584 | CA43 | *V. dahliae* | *Brassica oleracea* | cabbage | Gunma, Japan | - | Toshiyuki Usami, Hiroshi Sakai | 6 | *MAT1-2* |
| PD585 | CA26 | *V. dahliae* | *Brassica oleracea* | cabbage | Gunma, Japan | - | Toshiyuki Usami, Hiroshi Sakai | 6 | *MAT1-1* |
| PD587 | PoI | *V. dahliae* | *Papaver nudicaule* L. | Icelandic poppy | Chiba, Japan | - | Toshiyuki Usami | 6 | *MAT1-2* |
| PD588 | CA9 | *V. longisporum* d1 | *Brassica oleracea* | cabbage | Gunma, Japan | - | Toshiyuki Usami, Hiroshi Sakai | 6 | *MAT1-1* |
| PD589 | CA10 | *V. longisporum* d3 | *Brassica oleracea* | cabbage | Gunma, Japan | - | Toshiyuki Usami, Hiroshi Sakai | 6 | *MAT1-1* |
| PD590 | CA58 | *V. longisporum* d1 | *Brassica oleracea* | cabbage | Gunma, Japan | - | Toshiyuki Usami, Hiroshi Sakai | 6 | *MAT1-1* |
| PD591 | Dk-1 | *V. longisporum* d1 | *Raphanus sativus* L. | radish | Chiba, Japan | - | Toshiyuki Usami | 6 | *MAT1-1* |
| PD597 | 0701 | *V. dahliae* | *Solanum lycopersicum var. lycopersicum* | garden tomato | San Joaquin County, CA, USA | 2009 | Mike Davis | 1 | *MAT1-2* |
| PD598 | 0702 | *V. dahliae* | *Solanum lycopersicum var. lycopersicum* | garden tomato | San Joaquin County, CA, USA | 2009 | Mike Davis | 1 | *MAT1-2* |
| PD599 | 0703 | *V. dahliae* | *Gossypium hirsutum* | upland cotton | Tulare County, CA, USA | 2009 | Mike Davis | 1 | *MAT1-2* |
| PD600 | 0704 | *V. dahliae* | *Solanum lycopersicum var. lycopersicum* | garden tomato | Yolo County, CA, USA | 2009 | Mike Davis | 1 | *MAT1-2* |
| PD601 | 0705 | *V. dahliae* | *Solanum lycopersicum var. lycopersicum* | garden tomato | Yolo County, CA, USA | 2009 | Mike Davis | 1 | *MAT1-2* |
| PD602 | 0706 | *V. dahliae* | *Solanum lycopersicum var. lycopersicum* | garden tomato | Yolo County, CA, USA | 2009 | Mike Davis | 1 | *MAT1-2* |
| PD603 | 0707 | *V. dahliae* | *Solanum lycopersicum var. lycopersicum* | garden tomato | Yolo County, CA, USA | 2009 | Mike Davis | 1 | *MAT1-2* |
| PD604 | 0709 | *V. dahliae* | *Olea europaea* L. | olive | Tahama County, CA, USA | 2009 | Mike Davis, Ken Eden | 6 | *MAT1-2* |
| PD605 | 0710 | *V. dahliae* | *Gossypium hirsutum* | upland cotton | Merced County, CA, USA | 2009 | Mike Davis | 1 | *MAT1-2* |
| PD606 | 0711 | *V. dahliae* | *Citrullus lanatus var. lanatus* | watermelon | Stanislaus County, CA, USA | 2009 | Mike Davis | 1 | *MAT1-2* |
| PD607 | 0712 | *V. dahliae* | *Solanum lycopersicum var. lycopersicum* | garden tomato | Yolo County, CA, USA | 2009 | Mike Davis | 1 | *MAT1-2* |
| PD608 | 0713 | *V. dahliae* | *Solanum lycopersicum var. lycopersicum* | garden tomato | Yolo County, CA, USA | 2009 | Mike Davis | 1 | *MAT1-2* |
| PD609 | 0714 | *V. dahliae* | *Solanum lycopersicum var. lycopersicum* | garden tomato | Yolo County, CA, USA | 2009 | Mike Davis | 1 | *MAT1-2* |
| PD614 | MD73 | *V. longisporum* d3 | *Brassica napus* | rape | Germany | - | Dez Barbara | 6 | *MAT1-1* |
| PD615 | tom1 | *V. dahliae* | *Solanum lycopersicum var. lycopersicum* | garden tomato | Israel | - | Dez Barbara | 6 | *MAT1-2* |
| PD617 | P14 | *V. dahliae* | *Solanum lycopersicum var. lycopersicum* | garden tomato | Brazil | - | Dez Barbara | 6 | *MAT1-1* |
| PD620 | STR1 | *V. albo-atrum* | *Medicago sativa* | alfalfa | Canada | - | Dez Barbara | 6 | *MAT1-1* |
| PD621 | IMI 130213 | *V. nubilum* Pethybr. | - | - | UK | - | Dez Barbara | 6 | NA |
| PD622 | VDII | *V. longisporum* D1 | *Brassica napus* | rape | Germany | - | Dez Barbara | 1 | *MAT1-1* |
| PD623 | MD80 | *V. dahliae* | *Brassica napus* | rape | Germany | - | Dez Barbara | 6 | *MAT1-2* |
| PD624 | 90-10 | *V. longisporum* d1 | *Brassica oleracea var. botrytis* | cauliflower‡ | USA | - | Dez Barbara | 6 | *MAT1-1* |
| PD628 | ep4 | *V. dahliae* | *Solanum melongena* | eggplant | Israel | - | Dez Barbara | 6 | *MAT1-2* |
| PD629 | 004 | *V. longisporum* d2 | *Armoracia rusticana* | horseradish | IL | - | Dez Barbara | 6 | *MAT1-1* |
| PD630 | 84020 | *V. longisporum* d1 | *Brassica rapa var. rapa* L. | birdrape | Japan | - | Dez Barbara | 6 | *MAT1-1* |
| PD631 | V138I | *V. dahliae* | *Gossypium hirsutum* | upland cotton | Spain | - | Dez Barbara | 6 | *MAT1-2* |
| PD637 | Vd 73 | *V. dahliae* | *Linum usitatissimum* L. | common flax | Mecklenburg, Germany | - | Andreas von Tiedemann, K. Zeise | 6 | *MAT1-2* |
| PD638 | Vl 40 | *V. longisporum* d1 | *Brassica napus* | rape | Mecklenburg, Germany | - | Andreas von Tiedemann, K. Zeise | 6 | *MAT1-1* |
| PD640 | Chalons en Champs | *V. longisporum* d1 | *Brassica napus* | rape | France | - | Andreas von Tiedemann, Schaper | 6 | *MAT1-1* |
| PD641 | Vd 1 | *V. longisporum* D1 | *Brassica napus* | rape | Sweden | - | Andreas von Tiedemann | 1 | *MAT1-1* |
| PD642 | Vd 01:43 | *V. longisporum* D1 | *Brassica napus* | rape | Säbyholm, Sweden | - | Andreas von Tiedemann | 1 | *MAT1-1* |
| PD643 | Vd 02:50 | *V. longisporum* D1 | *Brassica napus* | rape | Kölbäck, Sweden | - | Andreas von Tiedemann | 1 | *MAT1-1* |
| PD644 | Vd 4 | *V. longisporum* d1 | *Brassica napus* | rape | Sweden | - | Andreas von Tiedemann | 6 | *MAT1-1* |
| PD645 | Vd 11 | *V. longisporum* D1 | *Brassica napus* | rape | Sweden | - | Andreas von Tiedemann | 1 | *MAT1-1* |
| PD649 | 0724.1 | *V. dahliae* | *Solanum tuberosum* | Irish potato | Siskiyou County, CA, USA | - | Mike Davis, Rob Wilson | 6 | *MAT1-2* |
| PD650 | 0724.2 | *V. dahliae* | *Solanum tuberosum* | Irish potato | Siskiyou County, CA, USA | - | Mike Davis, Rob Wilson | 1 | *MAT1-2* |
| PD651 | 0725.1 | *V. dahliae* | *Solanum lycopersicum var. lycopersicum* | garden tomato | Yolo County, CA, USA | - | Mike Davis, Gene Miyao | 1 | *MAT1-2* |
| PD652 | 0726.1 | *V. dahliae* | *Solanum lycopersicum var. lycopersicum* | garden tomato | Yolo County, CA, USA | - | Mike Davis, Gene Miyao | 1 | *MAT1-2* |
| PD656 | IRAN 453 C, IMI 090682 | *V. dahliae* | *Helianthus annuus* L. | annual sunflower | Canada | - | Rasoul Zare, W.E. Sackston | 6 | *MAT1-1* |
| PD660 | 730 C6 | *V. tricorpus* I. Isaac | *Lactuca sativa* | lettuce | California, USA | - | Subbarao Lab Collection | 6 | NA |
| PD663 | V.d.8, IPP 0094 | *V. dahliae* | *Solanum tuberosum* | Irish potato | Münsterland, Germany | 1997 | Andreas von Tiedemann, K. Zeise | 1 | *MAT1-2* |
| PD664 | V.d.9, IPP 0095 | *V. dahliae* | *Solanum tuberosum* | Irish potato | Brandenburg, Germany | 1995 | Andreas von Tiedemann, K. Zeise | 1 | *MAT1-2* |
| PD665 | V.d.16, IPP 0098 | *V. dahliae* | *Solanum tuberosum* | Irish potato | Mecklenburg, Germany | 1988 | Andreas von Tiedemann, K. Zeise | 1 | *MAT1-2* |
| PD666 | V.d.74, IPP 0111 | *V. dahliae* | *Helianthus annuus* | annual sunflower | Mecklenburg, Germany | 1994 | Andreas von Tiedemann, K. Zeise | 1 | *MAT1-2* |
| PD667 | V.D.85, IPP 0112 | *V. dahliae* | *Solanum tuberosum* | Irish potato | Mecklenburg, Germany | 1997 | Andreas von Tiedemann, K. Zeise | 1 | *MAT1-2* |
| PD668 | V.d.87, IPP 0113 | *V. dahliae* | *Solanum tuberosum* | Irish potato | Mecklenburg, Germany | 1997 | Andreas von Tiedemann, K. Zeise | 6 | *MAT1-2* |
| PD669 | V.d.88, IPP 0114 | *V. dahliae* | *Solanum tuberosum* | Irish potato | Mecklenburg, Germany | 1997 | Andreas von Tiedemann, K. Zeise | 6 | *MAT1-1* |
| PD671 | V1.2.3, IPP 0328 | *V. dahliae* | *Solanum tuberosum* | Irish potato | Wisconson, USA | - | Andreas von Tiedemann, Kelman | 1 | *MAT1-2* |
| PD672 | G29 | *V. longisporum* D1 | *Brassica napus* | rape | Friedrichsthal, Germany | 1990 | Milton Typas | 1 | *MAT1-1* |
| PD673 | F617-9 | *V. longisporum* D1 | *Brassica napus* | rape | Haute-Marne, France | 1988 | Milton Typas | 1 | *MAT1-1* |
| PD674 | G22 | *V. longisporum* d1 | *Brassica napus* | rape | Germany | 1990 | Milton Typas | 6 | *MAT1-1* |
| PD675 | F654-2 | *V. longisporum* D1 | *Brassica napus* | rape | Besançon, France | 1989 | Milton Typas | 1 | *MAT1-1* |
| PD676 | 161 | *V. longisporum* d1 | *Beta vulgaris* L. | sugar beet‡ | Sweden | 1981 | Milton Typas | 6 | *MAT1-1* |
| PD677 | 86207 | *V. longisporum* D1 | *Raphanus raphanistrum* L. | wild radish | Japan | 1980 | Milton Typas | 1 | *MAT1-1* |
| PD678 | 130 | *V. dahliae* | *Solanum lycopersicum var. lycopersicum* | garden tomato | Davis, CA, USA | 1976 | Milton Typas | 1 | *MAT1-2* |
| PD679 | 140 | *V. dahliae* | *Solanum melongena* | eggplant | Bari, Italy | 1976 | Milton Typas | 1 | *MAT1-2* |
| PD680 | G17 | *V. dahliae* | *Matthiola* Ait. f. | stock | Rostock, Germany | 1989 | Milton Typas | 6 | *MAT1-2* |
| PD681 | 220 | *V. albo-atrum* | *Medicago sativa* | alfalfa | USA | 1982 | Milton Typas | 1 | *MAT1-1* |
| PD682 | MAFF 235137 | *V. albo-atrum* | *Medicago sativa* | alfalfa | Hokkaido, Japan | 1981 | R. Sato, K. Kitazawa, obtained from NIAS Genebank, National Institute of Agrobiological Sciences, Japan | 1 | *MAT1-1* |
| PD687 | CBS 124.64, MUCL 9802 | *V. longisporum* d3 | *Armoracia rusticana* | horseradish | Niedersachsen, Germany | 1959 | C. Stark, obtained from CBS | 6 | *MAT1-1* |
| PD694 | IRAN 413 C | *V. dahliae* | *Pistacia vera* L. | pistachio nut | Iran | - | Rasoul Zare | 1 | *MAT1-2* |
| PD696 | IRAN 393 C | *V. dahliae* | *Pistacia vera* | pistachio nut | Kerman, Iran | - | Rasoul Zare, Aminaii & D. Ershad | 6 | *MAT1-2* |
| PD697 | IRAN 394 C | *V. dahliae* | *Pistacia vera* | pistachio nut | Rafsanjan, Iran | - | Rasoul Zare, Aminaii & D. Ershad | 1 | *MAT1-2* |
| PD698 | IRAN 434 C | *V. dahliae* | *Prunus dulcis* (P. Mill.) D.A. Webber | sweet almond | Eastern Azarbaijan, Iran | 2001 | Rasoul Zare | 6 | *MAT1-2* |
| PD701 | IRAN 667 C | *V. dahliae* | *Prunus armeniaca* L. | apricot | Shahrud, Iran | 2002 | Rasoul Zare | 1 | *MAT1-2* |
| PD704 | IRAN 1613 | *V. dahliae* | *Pistacia vera* | pistachio nut | Rafsanjan, Iran | 2009 | Rasoul Zare, F. Hassanzadeh | 1 | *MAT1-2* |
| PD705 | 54.1 | *V. dahliae* | *Chelidonium majus* L. | celandine | Stavropolsky kray, Russia | 2002 | Philipp Gannibal, Elena L. Gasich | 1 | *MAT1-2* |
| PD706 | 54.2 | *V. dahliae* | *Chelidonium majus* | celandine | Stavropolsky kray, Russia | 2002 | Philipp Gannibal, Elena L. Gasich | 6 | *MAT1-2* |
| PD707 | 54.4 | *V. dahliae* | *Tripleurospermum perforatum* (Mérat) M. Lainz | scentless false mayweed‡ | Novgorodskaya oblast, Savino, Russia | 2004 | Philipp Gannibal | 6 | PCRs failed |
| PD708 | 23.1 | *V. dahliae* | *Papaver somniferum* L. | opium poppy | Poltavska oblast, Ustimovka, Ukraine | 2003 | Philipp Gannibal, Elena L. Gasich | 6 | *MAT1-2* |
| PD713 | IPP 0117, V.l.18 | *V. longisporum* D1 | *Brassica napus* | rape | Mecklenburg, Germany | 1989 | Andreas von Tiedemann, K. Zeise | 1 | *MAT1-1* |
| PD715 | IPP 0119, V.l.32 | *V. longisporum* d3 | *Brassica napus* | rape | Mecklenburg, Germany | 1988 | Andreas von Tiedemann, K. Zeise | 6 | *MAT1-1* |
| PD716 | IPP 0126, V.l.84 | *V. longisporum* d1 | *Brassica napus* | rape | Mecklenburg, Germany | 1997 | Andreas von Tiedemann, K. Zeise | 6 | *MAT1-1* |
| PD717 | INRA 617, 091215 | *V. dahliae* | *Brassica napus* | rape | France | - | Christina Dixelius | 6 | *MAT1-1* |
| PD718 | INRA 591, 091215 | *V. dahliae* | *Brassica napus* | rape | France | - | Christina Dixelius | 6 | *MAT1-1* |
| PD719 | INRA 544, 091215 | *V. dahliae* | *Brassica napus* | rape | France | - | Christina Dixelius | 6 | *MAT1-1* |
| PD720 | 44-4 | *V. longisporum* d1 | *Brassica napus* | rape | Sweden | - | Christina Dixelius | 6 | *MAT1-1* |
| PD721 | 43-3 | *V. longisporum* d1 | *Brassica napus* | rape | Sweden | - | Christina Dixelius | 6 | *MAT1-1* |
| PD722 | VD12 | *V. longisporum* D1 | *Brassica napus* | rape | Sweden | - | Christina Dixelius | 1 | *MAT1-1* |
| PD723 | 40-2 | *V. longisporum* D1 | *Brassica napus* | rape | Sweden | - | Christina Dixelius | 1 | *MAT1-1* |
| PD724 | 42-1 | *V. longisporum* D1 | *Brassica napus* | rape | Sweden | - | Christina Dixelius | 1 | *MAT1-1* |
| PD725 | VD13 | *V. longisporum* D1 | *Brassica napus* | rape | Sweden | - | Christina Dixelius | 1 | *MAT1-1* |
| PD726 | 37-1 | *V. longisporum* D1 | *Brassica napus* | rape | Sweden | - | Christina Dixelius | 1 | *MAT1-1* |
| PD727 | SDV-I011-08 | *V. dahliae* | *Armoracia rusticana* | horseradish | Illinois, USA | - | Mohammad Babadoost | 6 | *MAT1-2* |
| PD728 | SDV-I022-08 | *V. dahliae* | *Armoracia rusticana* | horseradish | Illinois, USA | - | Mohammad Babadoost | 6 | *MAT1-2* |
| PD729 | SDV-I047-08 | *V. dahliae* | *Armoracia rusticana* | horseradish | Illinois, USA | - | Mohammad Babadoost | 6 | *MAT1-2* |
| PD730 | SDV-I066-08 | *V. longisporum* d2 | *Armoracia rusticana* | horseradish | Illinois, USA | - | Mohammad Babadoost | 6 | *MAT1-1* |

*For *V. longisporum* the genotype is given for each isolate following the epithet as d1, d2 or d3. Lower case indicates confirmation by DNA sequencing, upper case by PCR screening only.

†The common name follows www.ITIS.gov accessed on June 6, 2010, except indicated otherwise.

‡Common name differing from www.ITIS.gov accessed on June 6, 2010.
