# Supplementary material for: The Ascomycete Verticillium longisporum Is a Hybrid and a Plant Pathogen with an Expanded Host Range
Source: PLoS One. 2011 Mar 24;6(3):e18260. doi: 10.1371/journal.pone.0018260 (PMC3063834; doi:10.1371/journal.pone.0018260)
Supplement: Table S3 — Support values above 70 within Verticillium dahliae not given in Figure 2 . (DOC) [file pone.0018260.s012.doc]

| Representatives of clades | Bayesian | Parsimony | Likelihood |
| --- | --- | --- | --- |
| PD323 + PD729 | 100 | - | - |
| PD323 + PD729 + P332 + PD615 | 89 | - | - |
| PD323 + PD729 + P332 + PD615 + PD585 | 97 | - | - |
| PD322 + PD404 +PD323 + PD729+ P332 + PD615 + PD585 | 97 | - | - |
| PD717 + PD718 | 84 | - | - |
| PD331+PD337 | 76 | - | - |
