# Supplementary material for: The Ascomycete Verticillium longisporum Is a Hybrid and a Plant Pathogen with an Expanded Host Range
Source: PLoS One. 2011 Mar 24;6(3):e18260. doi: 10.1371/journal.pone.0018260 (PMC3063834; doi:10.1371/journal.pone.0018260)
Supplement: Table S4 — Loci used for phylogenetic analyses; details of chromosomal locations, lengths of amplicons, introns and intergenic spacers, and locus IDs are given with respect to the sequenced genome of V. dahliae strain PD322 on the Broad Institute website ( http://www.broadinstitute.org/annotation/genome/verticillium_dahliae/MultiHome.html , accessed February 10, 2009). (DOC) [file pone.0018260.s013.doc]

| Locus | Chromosome | Amplicon length, bp | Intron length, bp | Intergenic spacer length, bp | Locus ID |
| --- | --- | --- | --- | --- | --- |
| ITS | unknown | 579 | 0 | 311 | NA |
| *ACT* | unknown | 588 | 391 | 0 | VDAG_08445.1 |
| *EF* | 2 | 683 | 462 | 0 | VDAG_05517.1 |
| *GPD* | 5 | 727 | 202 | 0 | VDAG_08916.1 |
| *OX* | 8 | 710 | 157 | 0 | VDAG_06644.1 |
| *TS* | 1 | 604 | 210 | 0 | VDAG_01254.1 |
| *TUB* | 1 | 646 | 409 | 0 | VDAG_10074.1 |
| *MAT1-1* | 3† | 2372-2388* | 61 | 1521-1537 | NA |

**V. dahliae* strains PD502, PD585, PD404, PD617: were 2372, 2379, 2381, 2388 bp, respectively, and 1521, 1528, 1530, 1537 bp for intergenic spacers, respectively.

†Refers to position of *MAT1-2* idiomorph in *V. dahliae* strain PD322.
