# Supplementary material for: The Ascomycete Verticillium longisporum Is a Hybrid and a Plant Pathogen with an Expanded Host Range
Source: PLoS One. 2011 Mar 24;6(3):e18260. doi: 10.1371/journal.pone.0018260 (PMC3063834; doi:10.1371/journal.pone.0018260)
Supplement: Table S5 — The DNA sequences of all primers used are listed by primer name in ascending alphanumeric order. Primer sequences are given 5′->3′. (DOC) [file pone.0018260.s014.doc]

|  | 5'->3' |
| --- | --- |
| ActF2d1 | GATGCTCAAGCAGTACAC |
| ActF2d2 | GATGCTCAAGCAGTACAT |
| ActFa1 | CTCGATGCTCAAGCAGTGA |
| Alf | AGCGGAAAGCCATGAACGCATTC |
| ALF3 | AGCGAGGTAGGCCAGCAGGT |
| ALF4 | AGGGAGTGGGATGCGAGCCA |
| ALF5 | AAGACTGCTGCGTGCCTGCC |
| EFfa1 | GTGGAGCCCCGTATCTTGAAT |
| EFfd1 | TAAGTGGAGCCCTTGCTTGAT |
| EFfd2 | AAGTGGAGCCCCTGCTTGAA |
| GPDfa1 | TCTCTCCCGGCCGTGGTC |
| GPDfd1 | TCCCCGGCCTTGGTCTGAT |
| GPDfd2 | AGTAACCCCCCCCAAACCAAAGT |
| HMG21f | CGGCCGCCCAATTCGTACATCC |
| MAT11r | CAGTCAGATCCAACCTGCTGGCC |
| MAT12r | CGGGAGACGCACTCGCGAT |
| MAT12r3 | ACGGGAATGAGCCTGGCGGT |
| MAT12r4 | ACGTGTGAAGGTGCTTGCTGT |
| MAT12r5 | GGCAGGCACGCAGCAGTCTT |
| MAT21r | CATGCCTTCCATGCCATTAGTAGCC |
| MATa1f | GGTAGGGAGTGGGATGCG |
| MATa1r | TTTTAGCTCATTGTATTGCTCAA |
| MATdf | CTGGAGGTAGGGAGTGGGA |
| MATdr | TTTTAGCTCATTGTATTGCTCAT |
| OxFa1 | ATGCTCTTCCTGACCG |
| OxFd1 | TCTAGTATATTGAAGTCCTCA |
| OxFd2 | ACACCCTTTCGAGACG |
| TsF2d1 | GGAAGACATGACTGACATG |
| TsFa1 | CGCACGACGGCATTGC |
| TsFd2 | ACGACGGCATGGTCTC |
| VActf | TAATTCACAATGGAGGGTAGG |
| VActR | GTAAGGATACCACGCTTGG |
| VEFf | AACGTCGTCGTCATCGGCCACG |
| VEFr | CCACGCTCACGCTCGGCCTT |
| VGPDf | GGTCGCATTGTCTTCCGCAACG |
| VGPDf2 | GGCATCAACGGTTTCGGCC |
| VGPDr | GTAGGAGTGGACGGTGGTCATGAG |
| VOxF | ATGTCAACAACGCTGGGGT |
| VOxR | CGGCCTTGTAGATGGAAGTG |
| VTs2R | GGAACGAGACGGCCTCCT |
| VTs3f | GCGCTGCAAGGCCGAGAAC |
| VTs3r | GCGGAACGAGACGGCCTCC |
| VTsF | TTCAGCGCTGCAAGGCCG |
| VTsR | GCTTGCGGAACGAGACGGC |
| VTubF2 | CAAGATGCGTGAGATTGTAAGT |
| VTubR | AAGAGCTGGCCGAAGGGACC |
| ALf6 | GTGGGCTCTCGCGTCTTC |
| ALf7 | GAGTATTCCTGTTCTTCATG |
| MAT12r6 | AAATACTCGTATCATCTCTTG |
| MAT12r7 | AGCTCATTGTATTGCTCA |
| MAT12r8 | CCTCCGTCAAGACGCTGG |
