# Supplementary material for: The Ascomycete Verticillium longisporum Is a Hybrid and a Plant Pathogen with an Expanded Host Range
Source: PLoS One. 2011 Mar 24;6(3):e18260. doi: 10.1371/journal.pone.0018260 (PMC3063834; doi:10.1371/journal.pone.0018260)
Supplement: Table S6 — PCR conditions used in this study. For all loci, forward and reverse PCR primers, annealing temperature, annealing temperature for cloning, and expected product length in V. dahliae strain PD322 are given. For details on PCR conditions, see text. (DOC) [file pone.0018260.s015.doc]

| Locus | Primer (forward) | Primer (reverse) | Annealing temperature , °C | Annealing temperature cloning, °C | Amplicon length, bp |
| --- | --- | --- | --- | --- | --- |
| *ACT* | VActF | VActR | 48 | 53 | 588 |
| *EF* | VEFf | VEFr | 60 | 65 | 683 |
| *GPD* | VGPDf2 | VGPDr | 56 | NA | 727 |
| *GPD* | VGPDf | VGPDr | NA | 59 | 703 |
| *OX* | VOx3f | VOx2r | 54 | NA | 710 |
| *OX* | VOxf | VOxR | NA | 56 | 649 |
| *TS* | VTs3f | VTs3r | 59 | NA | 604 |
| *TS* | VTsf | VTsR | NA | 63 | 612 |
| *ITS* | ITS1-F | ITS4 | 51 | 51 | 579 |
| *MAT1-1** | Alf | MAT12r | 59 | 59 | 2297 |
| *MAT1-1*† | Alf | MAT11r | 57 | NA | 291 |
| *MAT1-2*† | HMG21f | MAT21r | 57 | NA | 330 |
| *TUB* | VTubF2 | VTubR | 52 | 58 | 646 |

*PCR extension time is 2.5 minutes; *V. dahliae* strain PD404 was sequenced with primers Alf, Alf3, Alf4, Alf5, Alf6, Alf7, MAT12r, MAT12r3, MAT12r4, MAT12r5, MAT12r6, MAT12r7, PD585 and *V. dahliae* strain PD617 additionally with MAT12r8; amplicons length based on *V. albo-atrum* strain PD338.

†Primers used to screen for presence of *MAT1-1* and *MAT1-2* in *V. dahliae* and *V. longisporum*.
