# Supplementary material for: The Ascomycete Verticillium longisporum Is a Hybrid and a Plant Pathogen with an Expanded Host Range
Source: PLoS One. 2011 Mar 24;6(3):e18260. doi: 10.1371/journal.pone.0018260 (PMC3063834; doi:10.1371/journal.pone.0018260)
Supplement: Table S7 — Loci and conditions used for V. longisporum allele-specific PCR amplifications. For each locus and allele, forward and reverse primers, annealing temperature, amplicon length with respect to V. dahliae strain PD322, numbers of introns targeted and total intron lengths are given. For details on PCR conditions, see text. (DOC) [file pone.0018260.s016.doc]

| Locus | Alleles amplified | Primer (forward) | Primer (reverse) | Annealing temperature, °C | Amplicon length, bp | Total intron length, bp | Introns number |
| --- | --- | --- | --- | --- | --- | --- | --- |
| *Act* | A1 | ActFa1 | VActR | 52 | 506 | 316 | 3 |
| *Act* | D1 | ActF2d1 | VActR | 55 | 503 | 313 | 3 |
| *Act* | D2/D3 | ActF2d2 | VActR | 55 | 503 | 313 | 3 |
| *EF* | A1 | EFfa1 | VEFr | 62 | 574 | 438 | 2 |
| *EF* | D1 | EFfd1 | VEFr | 62 | 577 | 441 | 2 |
| *EF* | D2/D3 | EFfd2 | VEFr | 62 | 576 | 440 | 2 |
| *GPD* | A1 | GPDfa1 | VGPDr | 62 | 544 | 130 | 1 |
| *GPD* | D1 | GPDfd1 | VGPDr | 62 | 549 | 135 | 1 |
| *GPD* | D2/D3 | GPDfd2 | VGPDr | 62 | 612 | 198 | 1 |
| *MAT1-1** | A1 | MATa1f | MATa1r | 50 | 419 | 0 | 0 |
| *MAT1-1** | D1/D2/D3 | MATdf | MATdr | 50 | 419 | 0 | 0 |
| *OX* | A1 | OxFa1 | VOxR | 55 | 592 | 113 | 2 |
| *OX* | D1 | OxFd1 | VOxR | 55 | 450 | 46 | 1 |
| *OX* | D2/D3 | OxFd2 | VOxR | 55 | 505 | 101 | 1 |
| *TS* | A1 | TsFa1 | VTs2R | 56 | 514 | 185 | 2 |
| *TS* | D1 | TsF2d1 | VTs2R | 56 | 469 | 140 | 2 |
| *TS* | D2/D3 | TsFd2 | VTs2R | 56 | 511 | 182 | 2 |

**MAT1-1*: Amplicon length is given with respect to *V. albo-atrum* strain PD338. Amplicon is located in intergenic spacer between *MAT1-1-1* and *MAT1-1-2*.
